# Supplementary figures and images for: ATP binding facilitates target search of SWR1 chromatin remodeler by promoting one-dimensional diffusion on DNA
Source: eLife. 2022 Jul 25;11:e77352. doi: 10.7554/eLife.77352 (PMC9365391; doi:10.7554/eLife.77352)

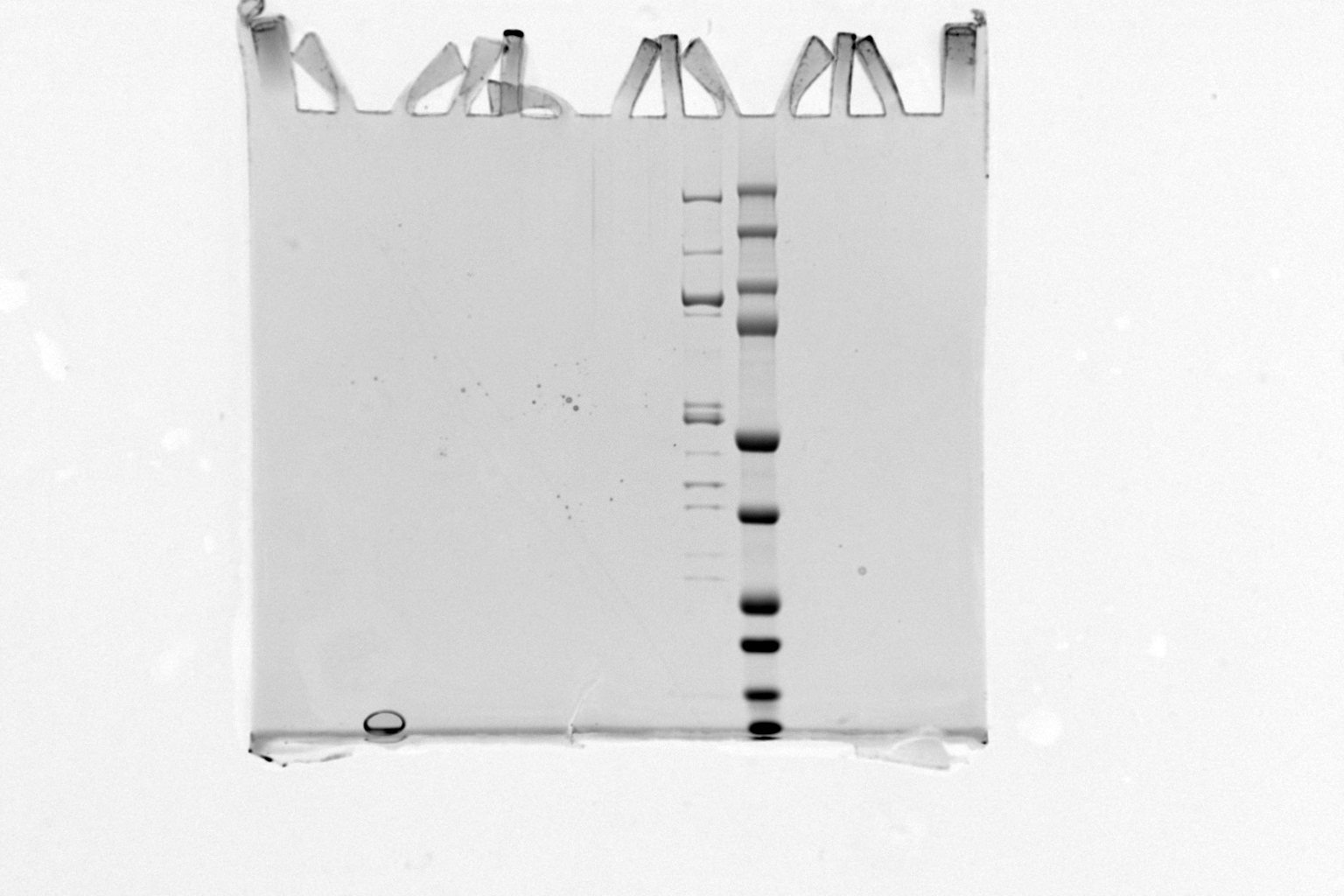

Supplement: Figure 1—source data 2. [file elife-77352-fig1-data2.zip › Figure 1 Source Data 2 Gels/cy3swr1v2[Coomassie].gel]

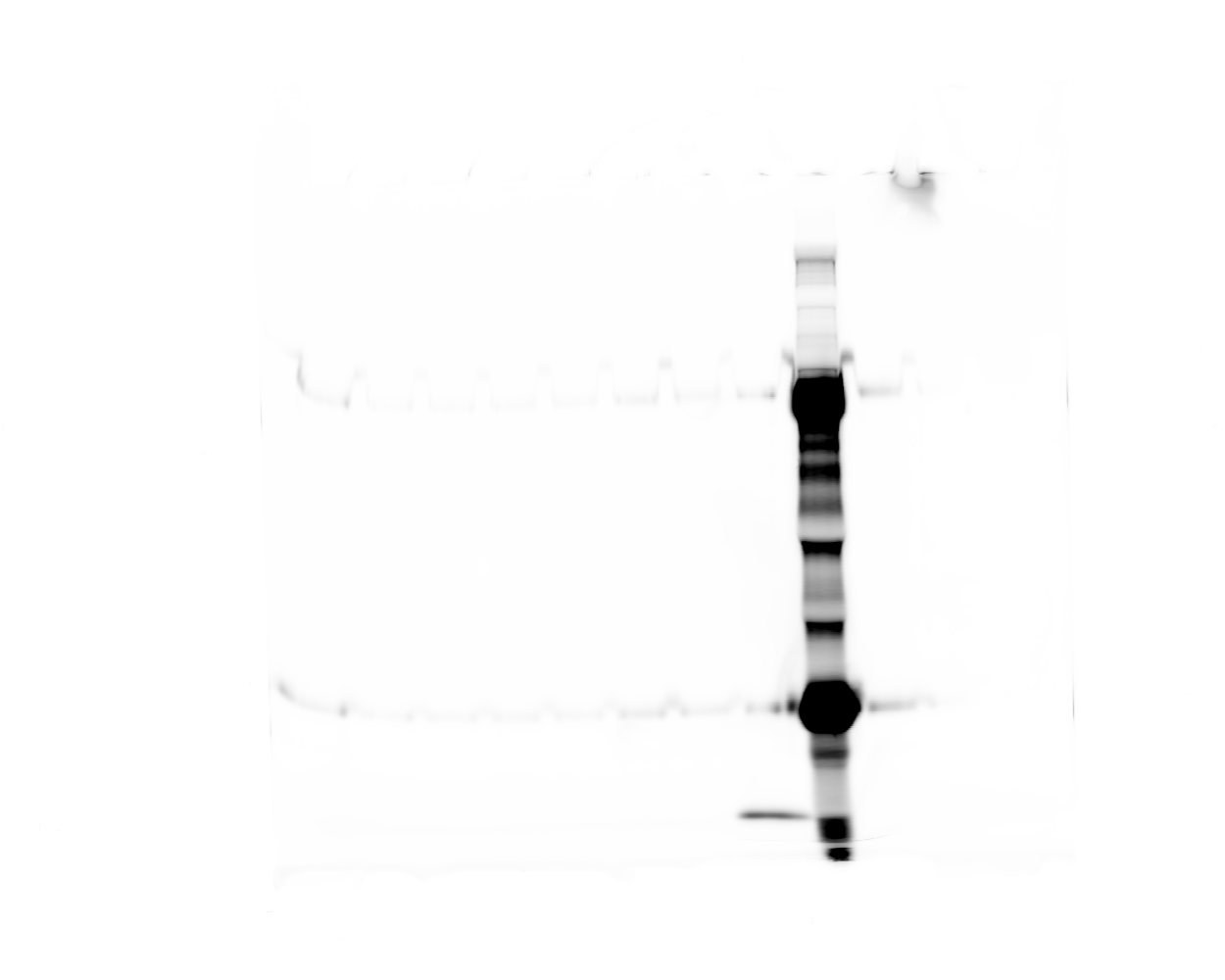

Supplement: Figure 1—source data 2. [file elife-77352-fig1-data2.zip › Figure 1 Source Data 2 Gels/cy3swr1v2-[Cy3].tif]

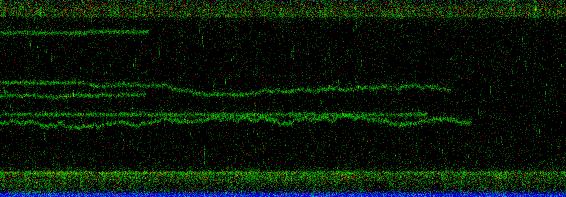

Supplement: Figure 2—source data 2. [file elife-77352-fig2-data2.zip › Figure 2 Source Data 2/20190829-201214 Kymograph 10(contrast corrected).tif]

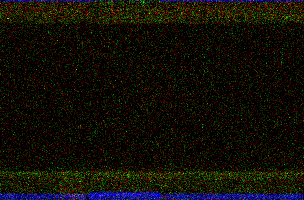

Supplement: Figure 2—figure supplement 1—source data 1. [file elife-77352-fig2-figsupp1-data1.zip › Figure 2 figure supplement 1/kymo_2.tiff (RGB).tif]

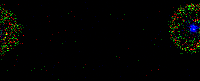

Supplement: Figure 2—figure supplement 1—source data 1. [file elife-77352-fig2-figsupp1-data1.zip › Figure 2 figure supplement 1/scan_3-1-0002.tif]

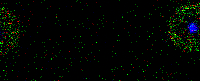

Supplement: Figure 2—figure supplement 1—source data 1. [file elife-77352-fig2-figsupp1-data1.zip › Figure 2 figure supplement 1/scan_3-1-0023.tif]

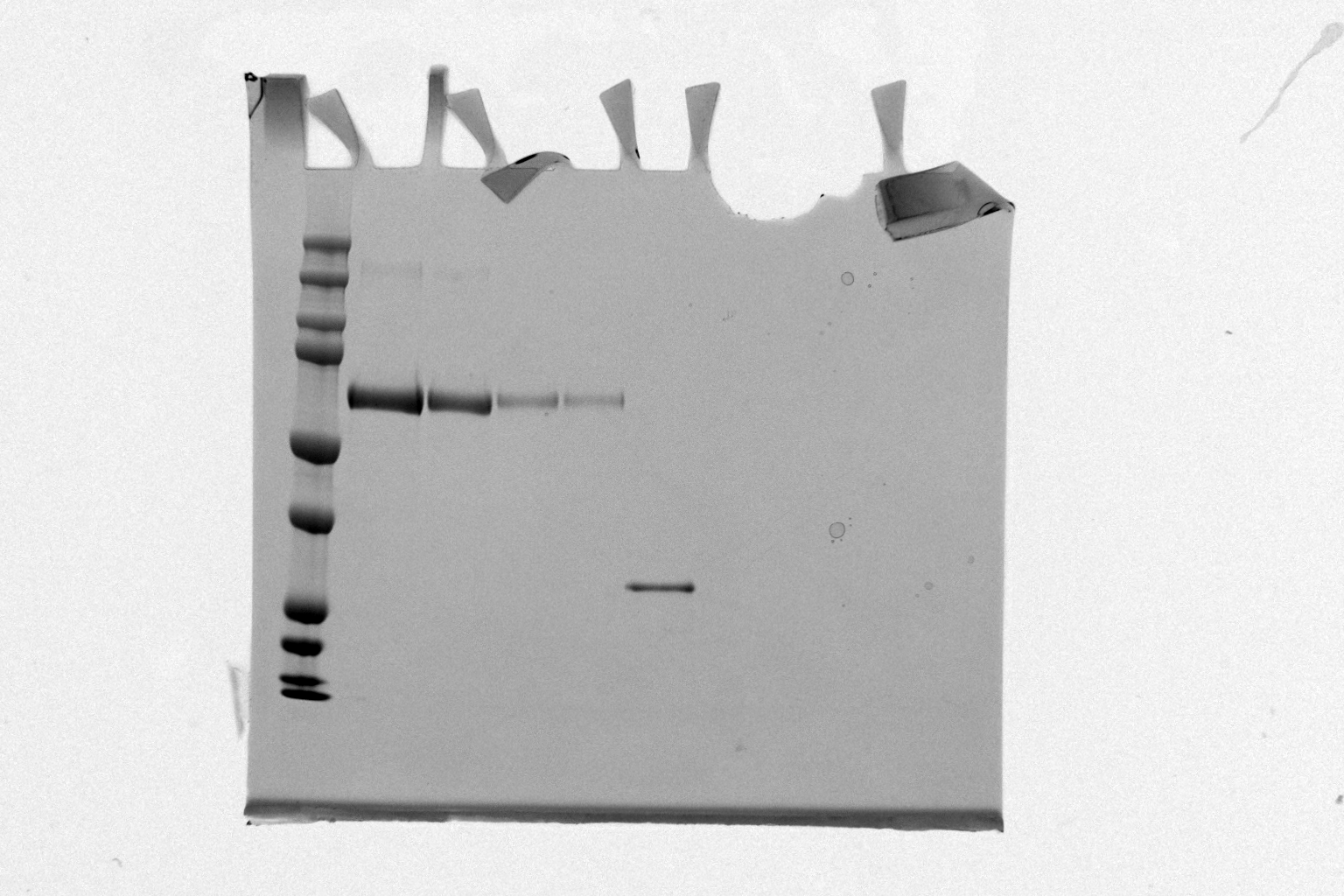

Supplement: Figure 4—figure supplement 1—source data 1. [file elife-77352-fig4-figsupp1-data1.zip › Figure 4 figure supplement 1 Gels/coomassie_scan.gel]

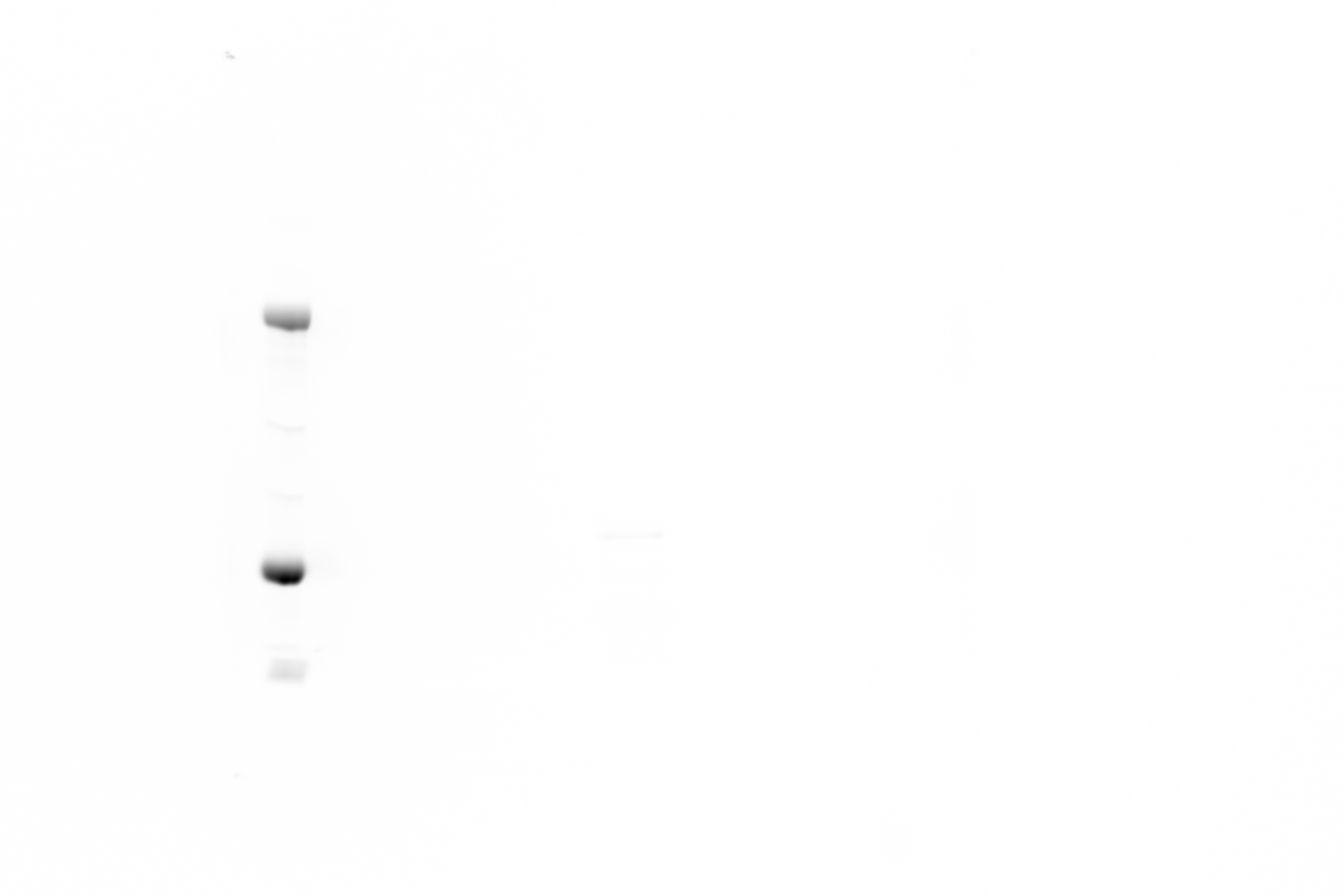

Supplement: Figure 4—figure supplement 1—source data 1. [file elife-77352-fig4-figsupp1-data1.zip › Figure 4 figure supplement 1 Gels/cy3_scan.gel]

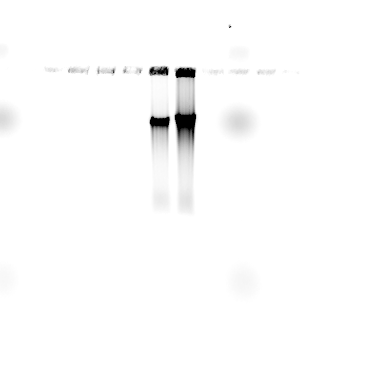

Supplement: Figure 6—figure supplement 1—source data 1. [file elife-77352-fig6-figsupp1-data1.zip › Figure 6 figure supplement 1 Gels/scan 2-[Cy5].png]

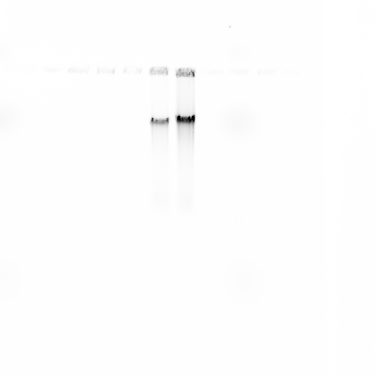

Supplement: Figure 6—figure supplement 1—source data 1. [file elife-77352-fig6-figsupp1-data1.zip › Figure 6 figure supplement 1 Gels/scan 2-[Cy5].tif]

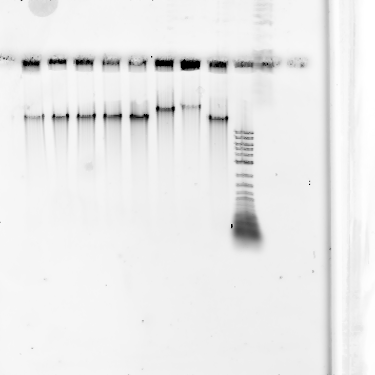

Supplement: Figure 6—figure supplement 1—source data 1. [file elife-77352-fig6-figsupp1-data1.zip › Figure 6 figure supplement 1 Gels/scan 2-[SYBR Gold].png]

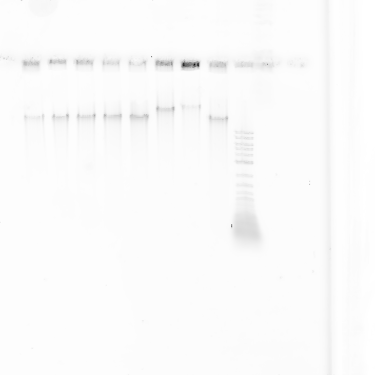

Supplement: Figure 6—figure supplement 1—source data 1. [file elife-77352-fig6-figsupp1-data1.zip › Figure 6 figure supplement 1 Gels/scan 2-[SYBR Gold].tif]
