# Supplementary material for: ATP binding facilitates target search of SWR1 chromatin remodeler by promoting one-dimensional diffusion on DNA
Source: eLife. 2022 Jul 25;11:e77352. doi: 10.7554/eLife.77352 (PMC9365391; doi:10.7554/eLife.77352)
Supplement: Figure 1—figure supplement 1—source data 1. [file elife-77352-fig1-figsupp1-data1.zip › Figure 1 figure supplement 1 Gels/Gel Images.pptx]

## Slide 1
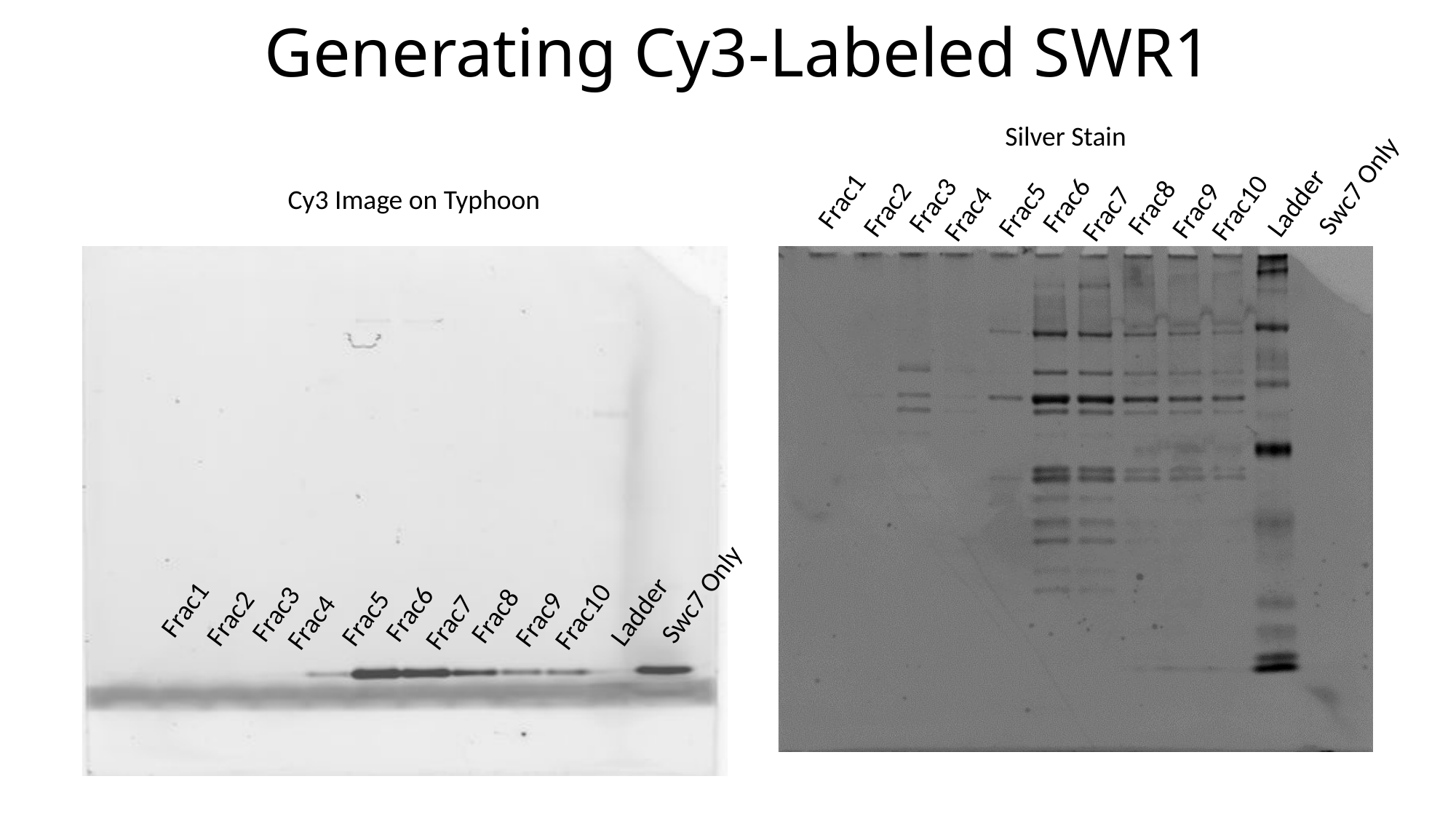

# Generating Cy3-Labeled SWR1
Silver Stain
Frac1
Frac6
Frac3
Frac8
Swc7 Only
Frac5
Ladder
Frac2
Frac9
Frac10
Frac7
Frac4
Cy3 Image on Typhoon
Frac1
Frac6
Frac3
Frac8
Swc7 Only
Frac5
Ladder
Frac2
Frac9
Frac10
Frac7
Frac4

## Slide 2
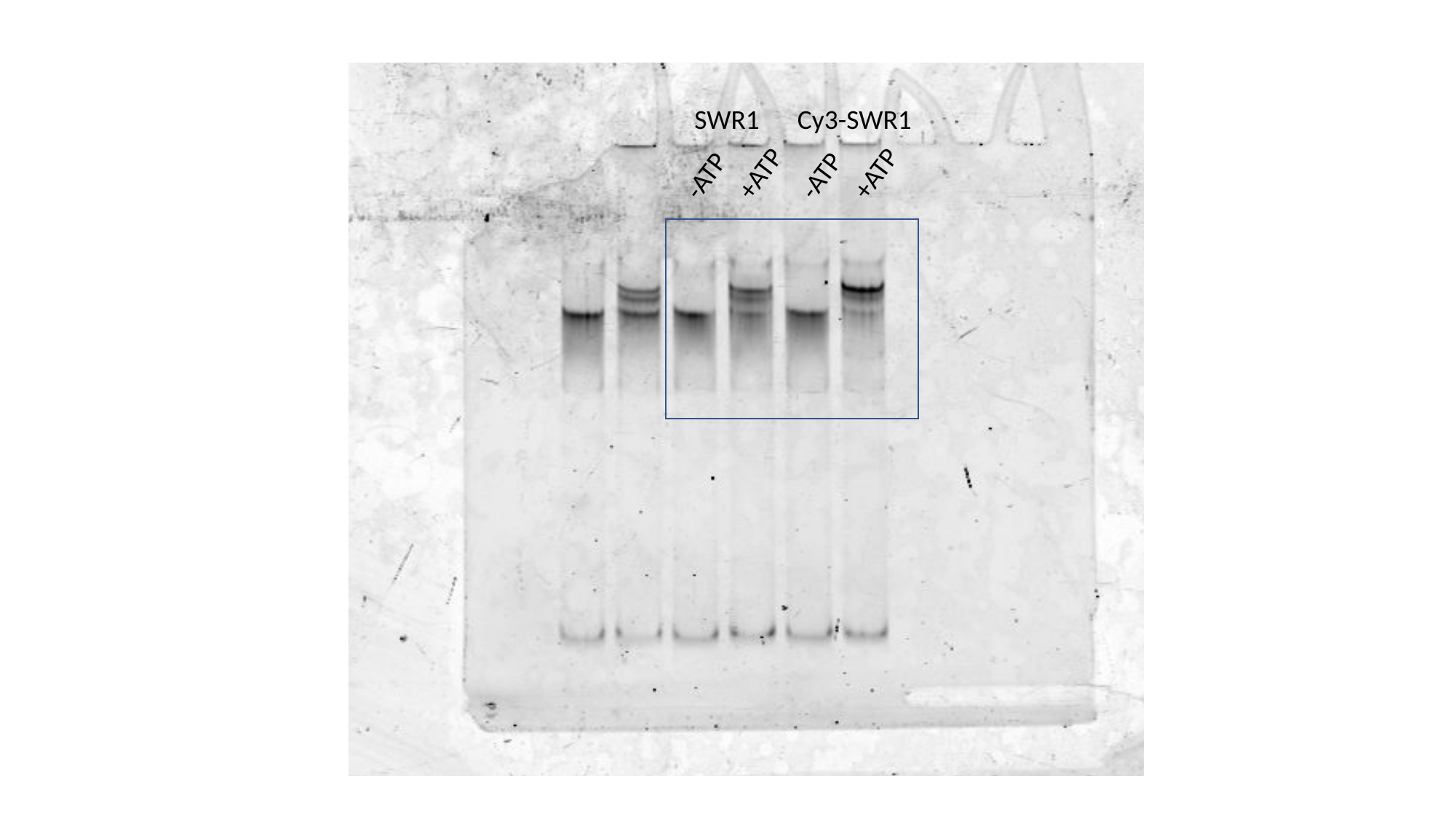

SWR1
Cy3-SWR1
-ATP
+ATP
-ATP
+ATP
