# Supplementary material for: ATP binding facilitates target search of SWR1 chromatin remodeler by promoting one-dimensional diffusion on DNA
Source: eLife. 2022 Jul 25;11:e77352. doi: 10.7554/eLife.77352 (PMC9365391; doi:10.7554/eLife.77352)
Supplement: Figure 5—source data 1. [file elife-77352-fig5-data1.zip › Figure 5 Source Data 1/Figure 5 All Colocalization Examples with Classifications Indicated.pdf]

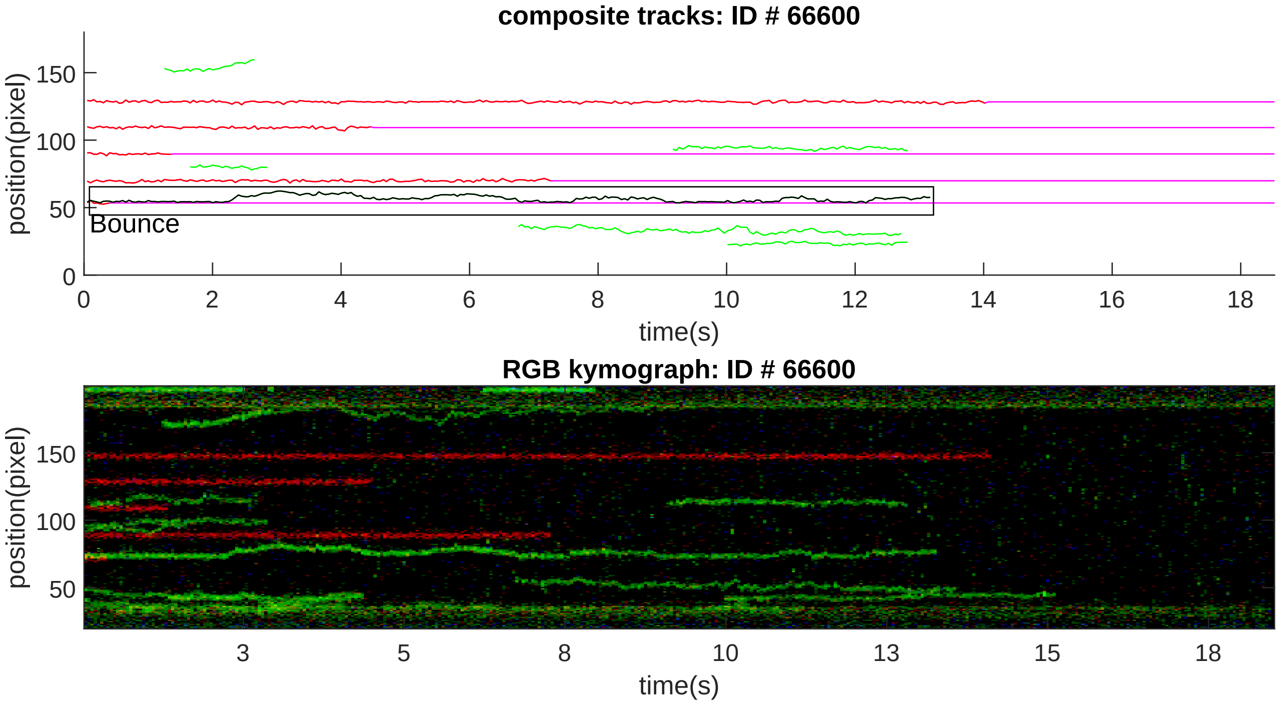

composite tracks: ID # 66600
position(pixel)
0
50
100
150
Bounce
time(s)
0
2
4
6
8
10
12
14
16
18
RGB kymograph: ID # 66600
position(pixel)
50
100
150
time(s)
3
5
8
10
13
15
18

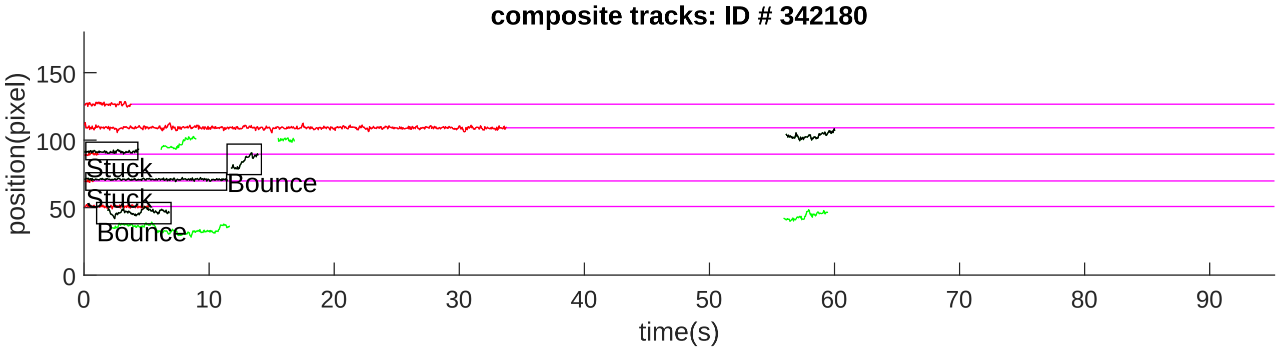

composite tracks: ID # 342180
position(pixel)
150
100
50
0
Stuck
Stuck
Bounce
Bounce
0
10
20
30
40
50
60
70
80
90
time(s)

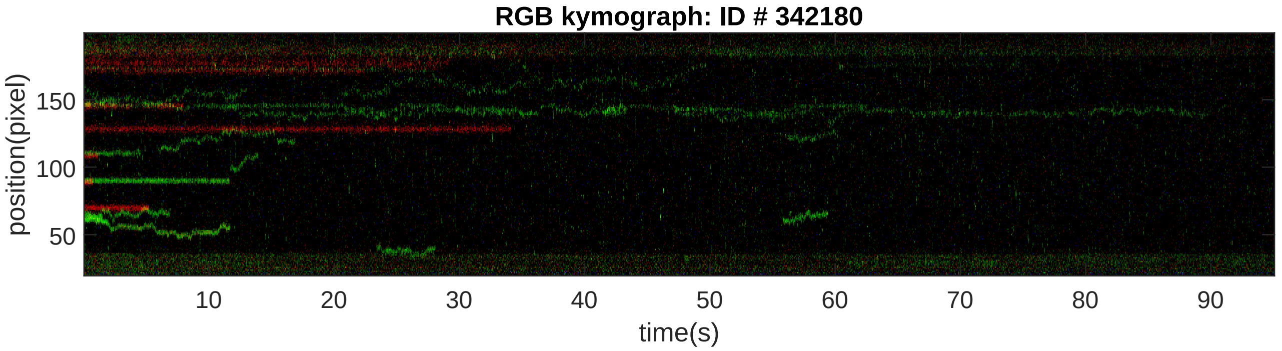

RGB kymograph: ID # 342180
position(pixel)
150
100
50
10
20
30
40
50
60
70
80
90
time(s)

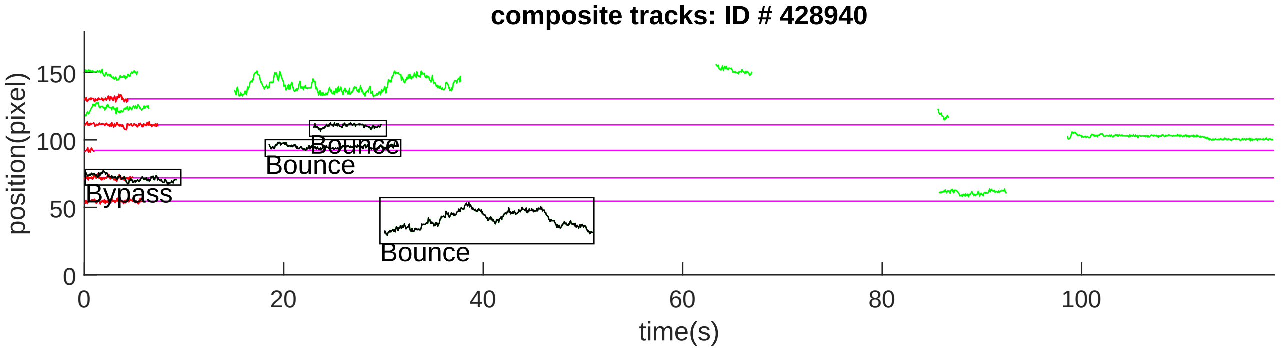
composite tracks: ID # 428940
position(pixel)
time(s)
Bounce
Bounce
Bounce
Bypass
Bounce
0
50
100
150
0
20
40
60
80
100

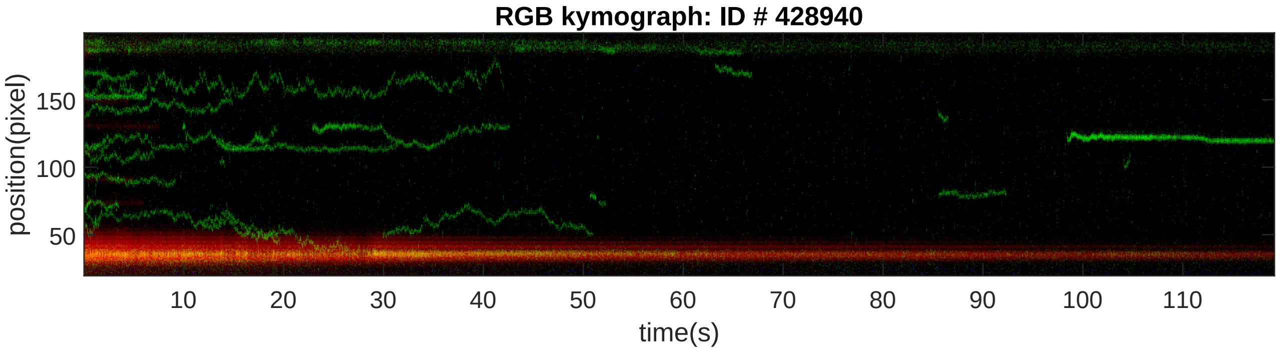
RGB kymograph: ID # 428940
position(pixel)
time(s)
50
100
150
10
20
30
40
50
60
70
80
90
100
110

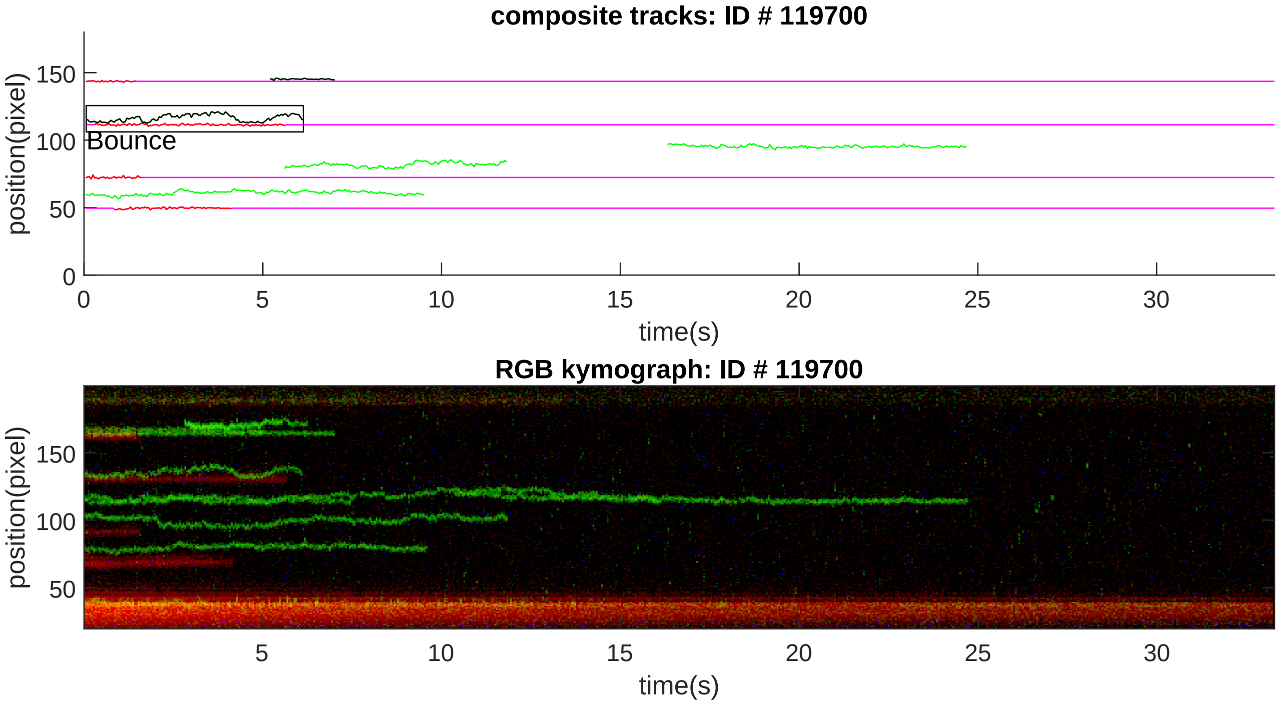
composite tracks: ID # 119700
position(pixel)
time(s)
Bounce
RGB kymograph: ID # 119700
position(pixel)
time(s)

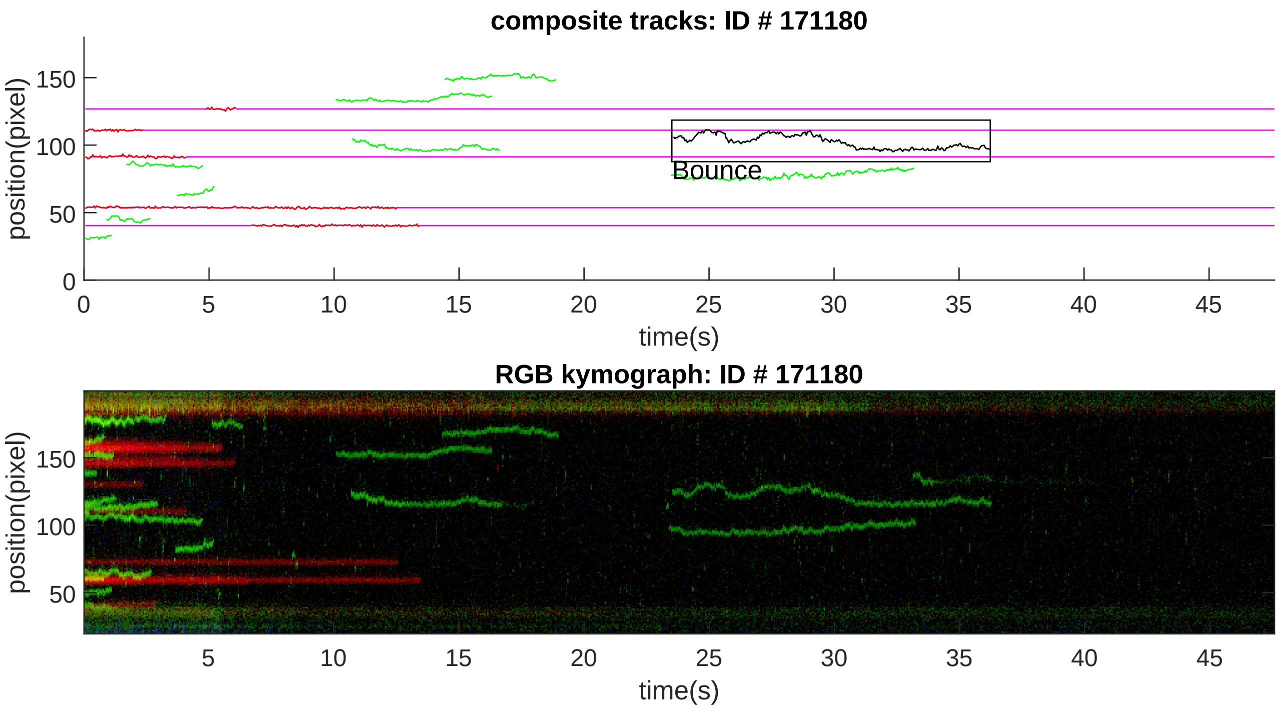
composite tracks: ID # 171180
position(pixel)
time(s)
Bounce
RGB kymograph: ID # 171180
position(pixel)
time(s)

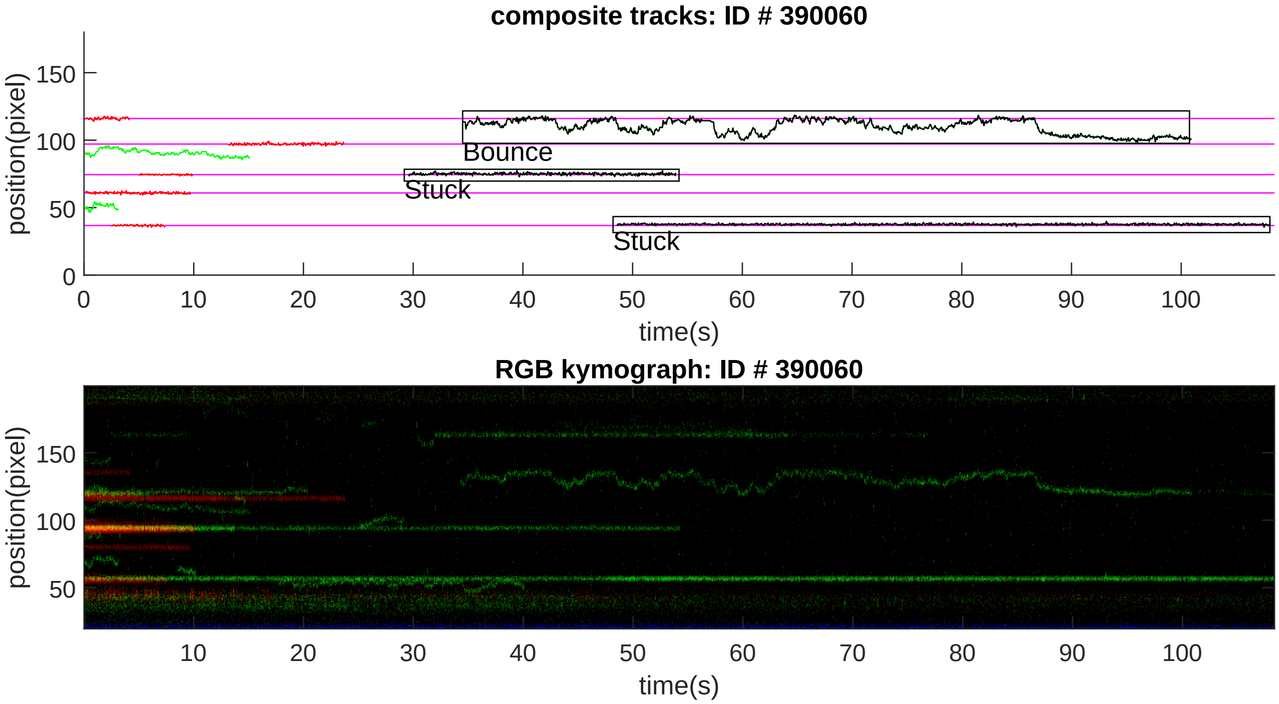
composite tracks: ID # 390060
position(pixel)
time(s)
Bounce
Stuck
Stuck
RGB kymograph: ID # 390060
position(pixel)
time(s)

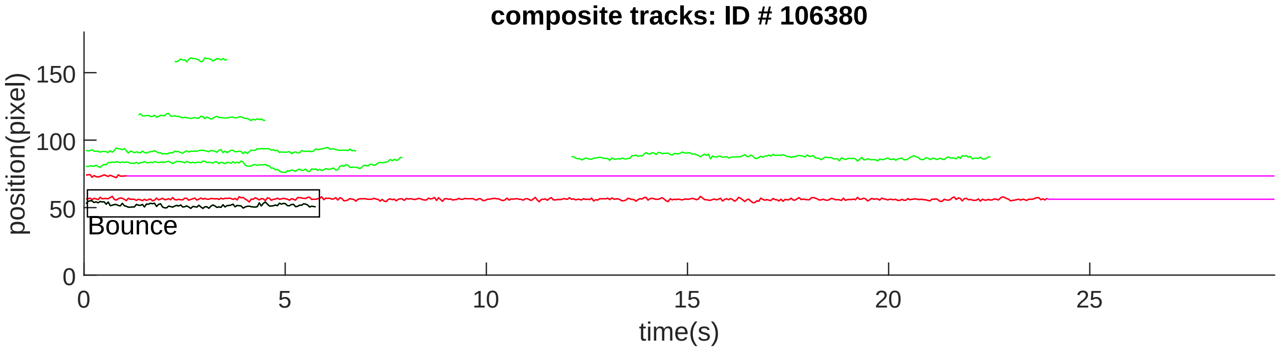
composite tracks: ID # 106380
position(pixel)
150
100
50
0
Bounce
0
5
10
15
20
25
time(s)

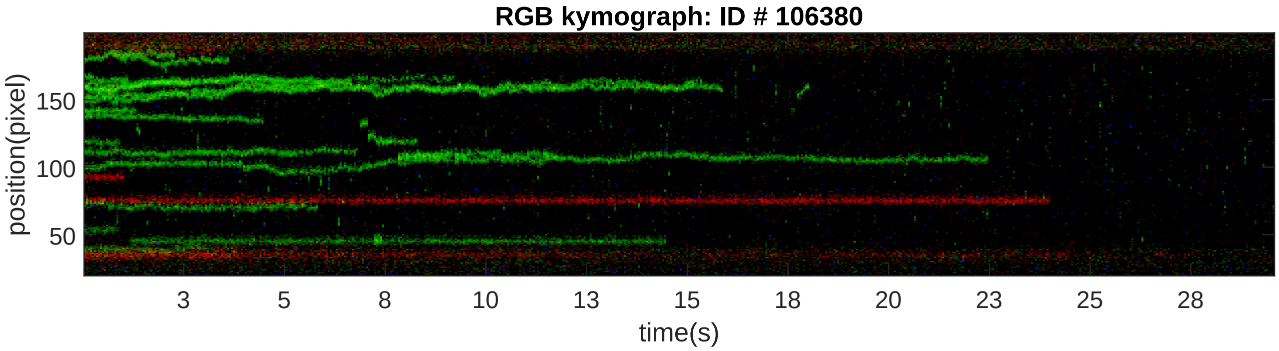
RGB kymograph: ID # 106380
position(pixel)
150
100
50
3
5
8
10
13
15
18
20
23
25
28
time(s)

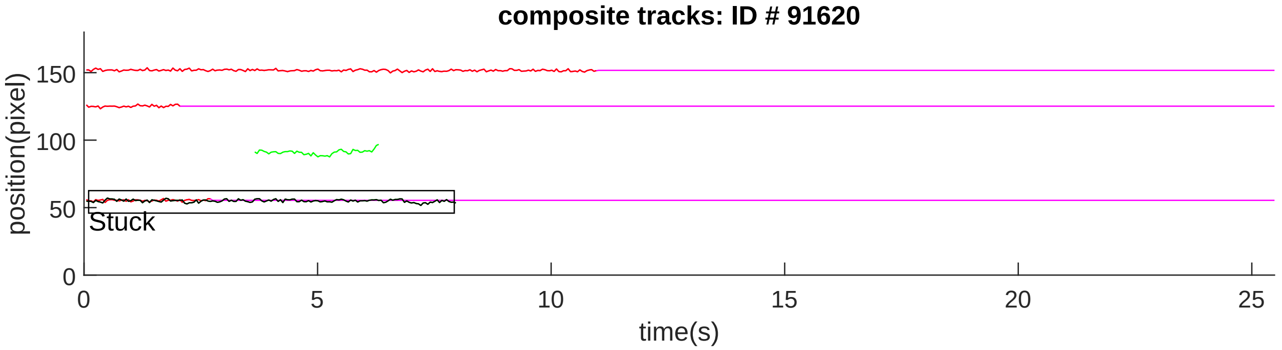

composite tracks: ID # 91620
position(pixel)
150
100
50
0
Stuck
0
5
10
15
20
25
time(s)

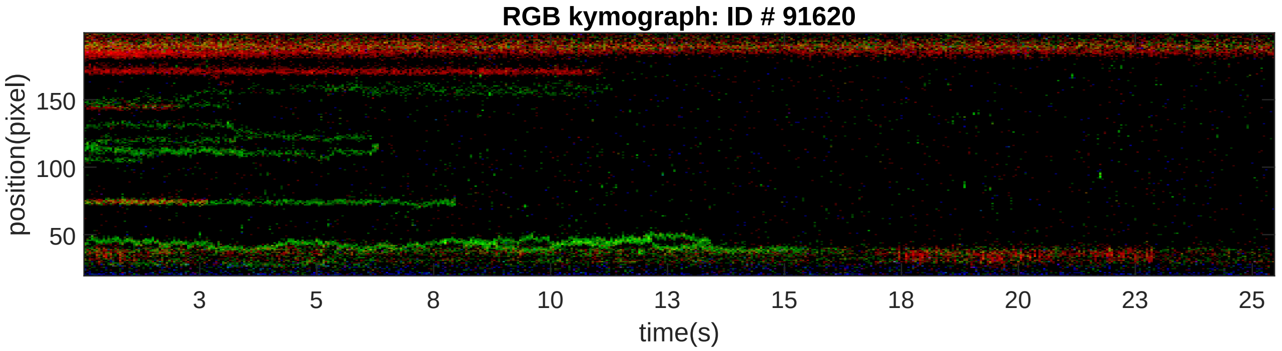

RGB kymograph: ID # 91620
position(pixel)
150
100
50
3
5
8
10
13
15
18
20
23
25
time(s)

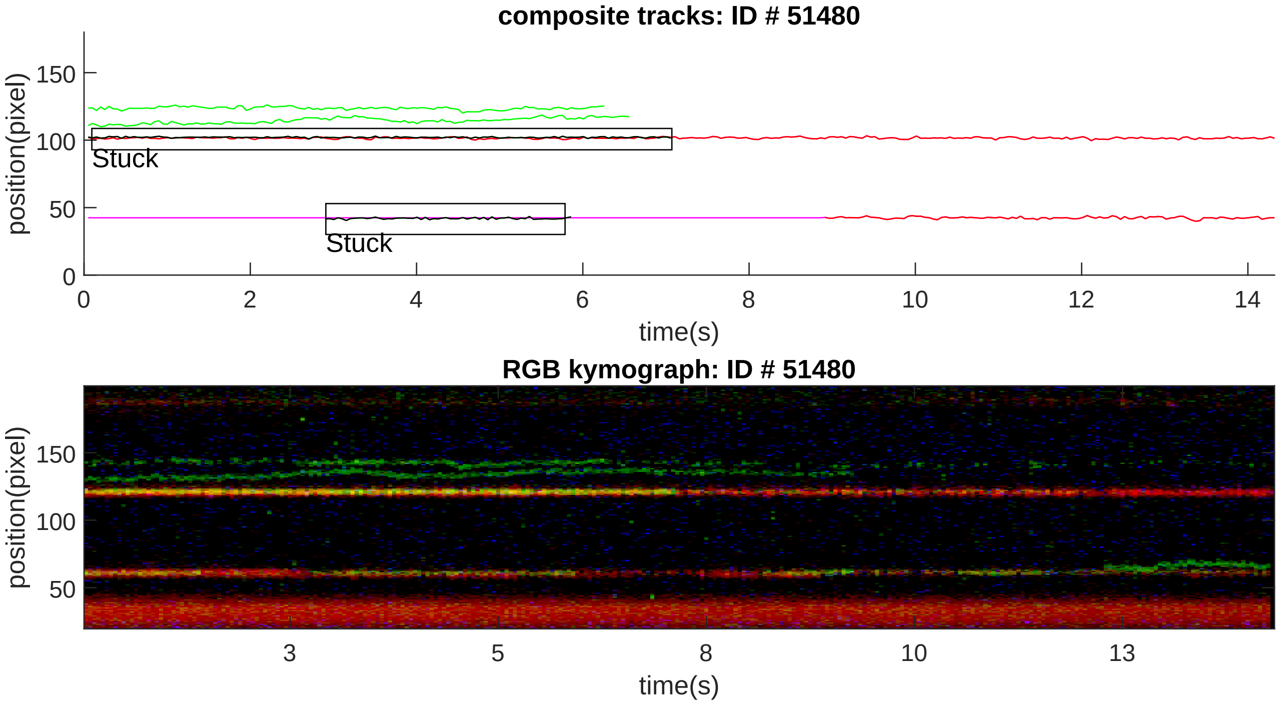
composite tracks: ID # 51480
position(pixel)
150
100
50
0
Stuck
Stuck
0
2
4
6
8
10
12
14
time(s)
RGB kymograph: ID # 51480
position(pixel)
150
100
50
3
5
8
10
13
time(s)

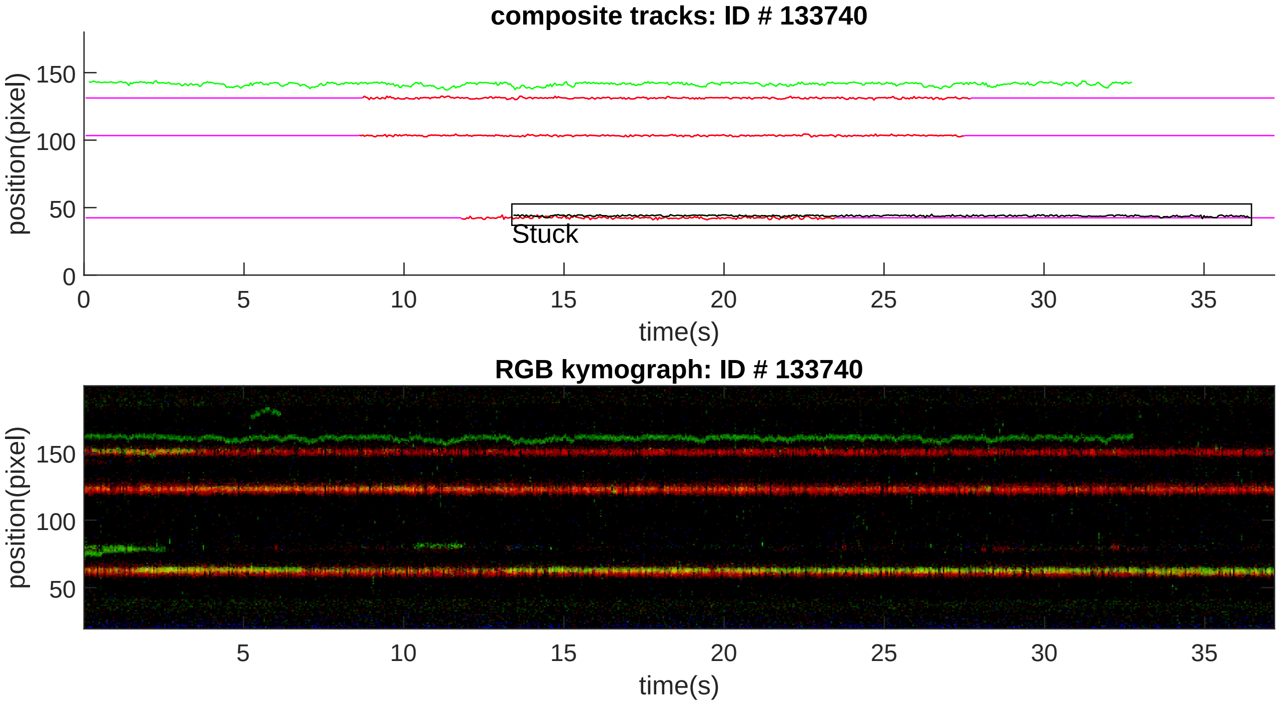
composite tracks: ID # 133740
position(pixel)
0
50
100
150
Stuck
0
5
10
15
20
25
30
35
time(s)
RGB kymograph: ID # 133740
position(pixel)
50
100
150
5
10
15
20
25
30
35
time(s)

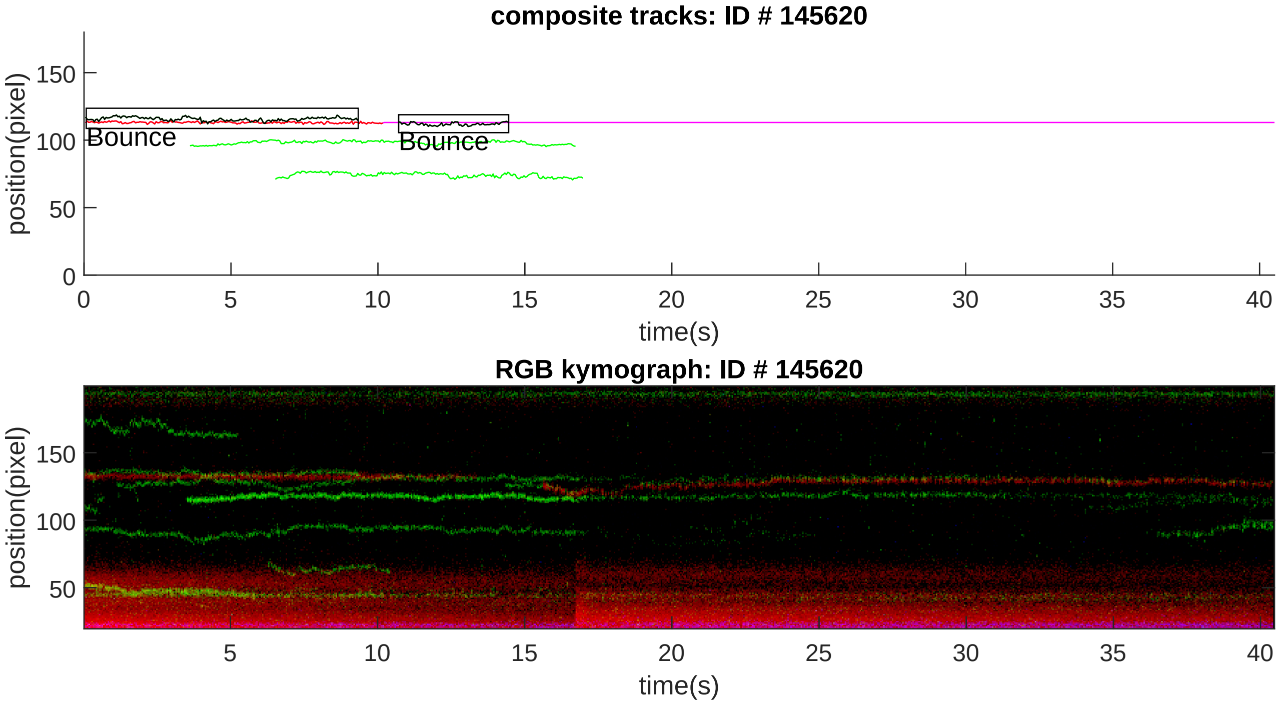
composite tracks: ID # 145620
position(pixel)
time(s)
Bounce
Bounce
RGB kymograph: ID # 145620
position(pixel)
time(s)

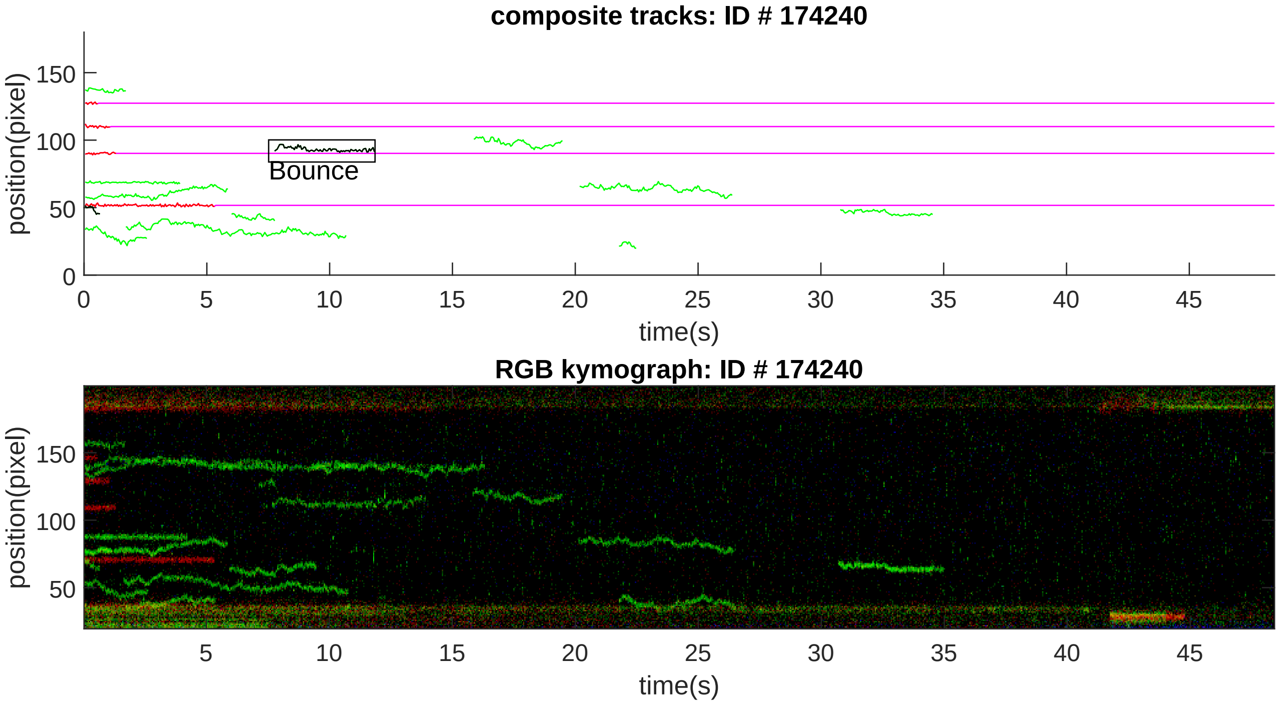
composite tracks: ID # 174240
position(pixel)
time(s)
Bounce
RGB kymograph: ID # 174240
position(pixel)
time(s)

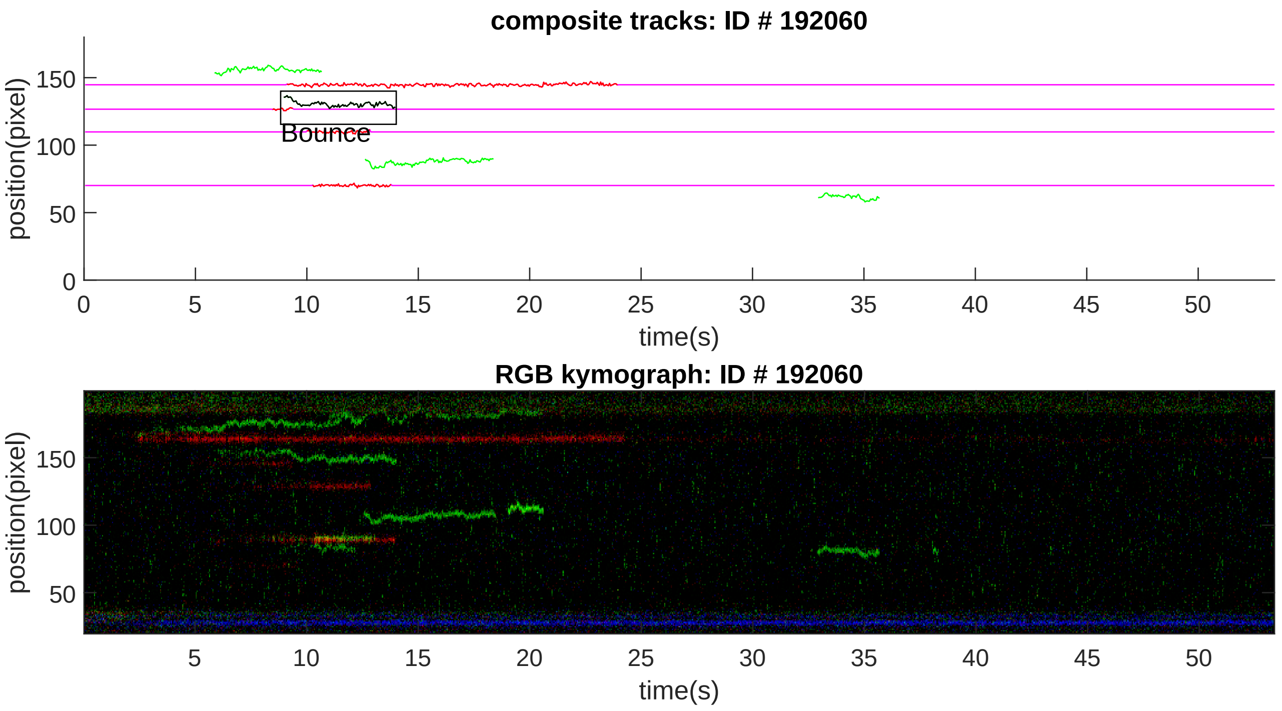

composite tracks: ID # 192060
position(pixel)
time(s)
Bounce
RGB kymograph: ID # 192060
position(pixel)
time(s)

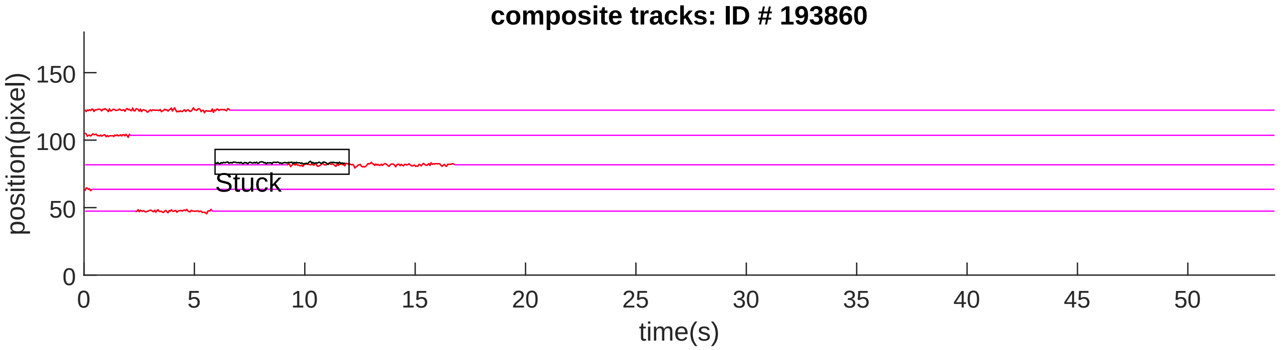
composite tracks: ID # 193860
position(pixel)
time(s)
Stuck
0
50
100
150
0
5
10
15
20
25
30
35
40
45
50

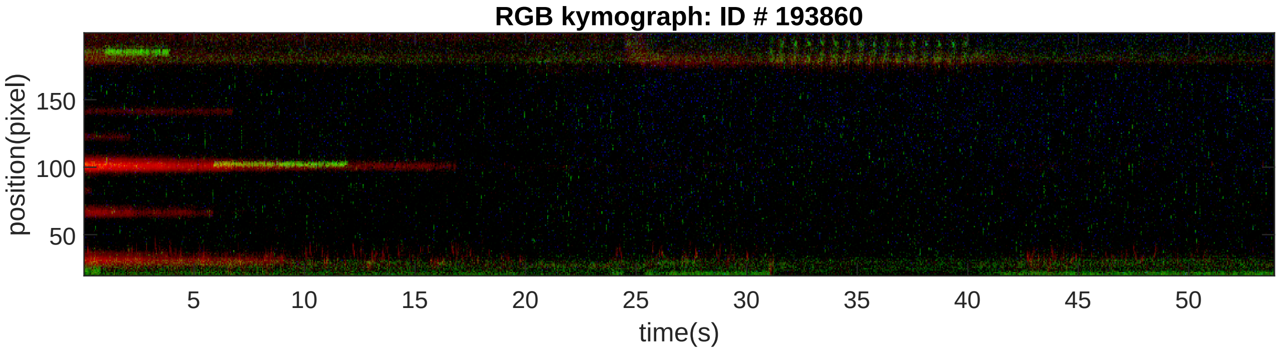
RGB kymograph: ID # 193860
position(pixel)
time(s)
50
100
150
5
10
15
20
25
30
35
40
45
50

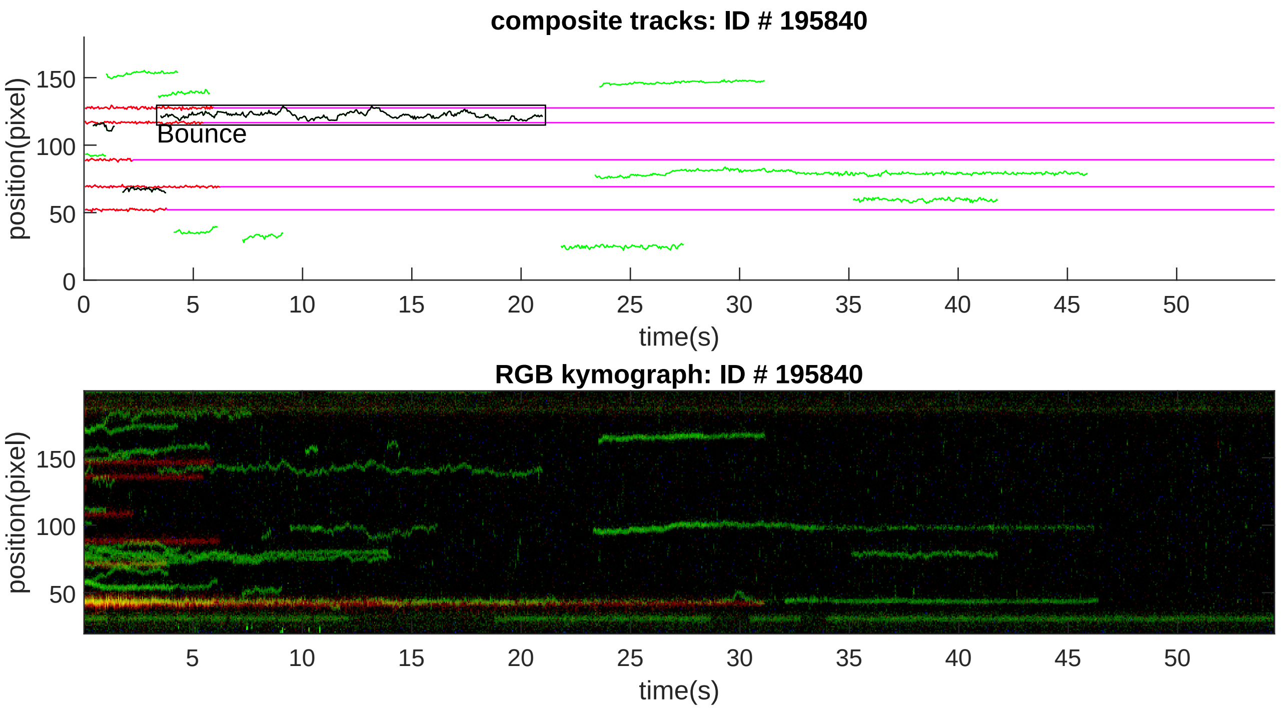

composite tracks: ID # 195840
position(pixel)
Bounce
time(s)
RGB kymograph: ID # 195840
position(pixel)
time(s)

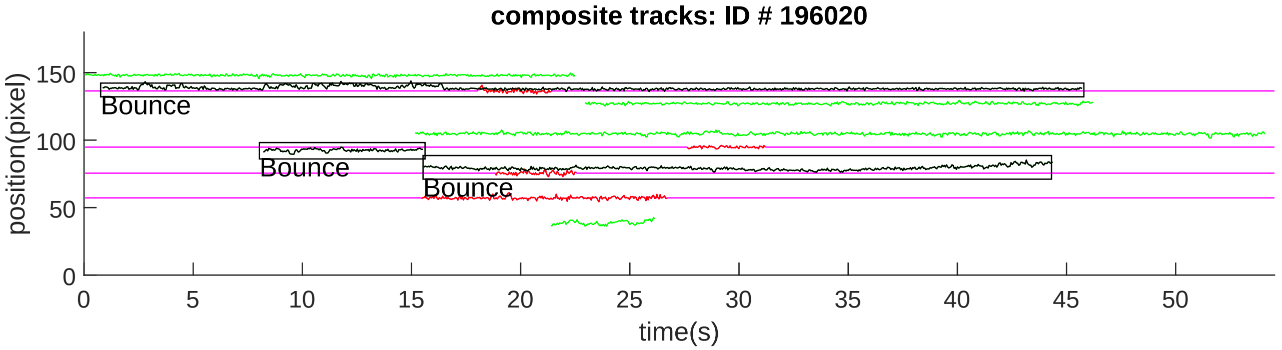
composite tracks: ID # 196020
position(pixel)
time(s)
Bounce
Bounce
Bounce
0
50
100
150
0
5
10
15
20
25
30
35
40
45
50

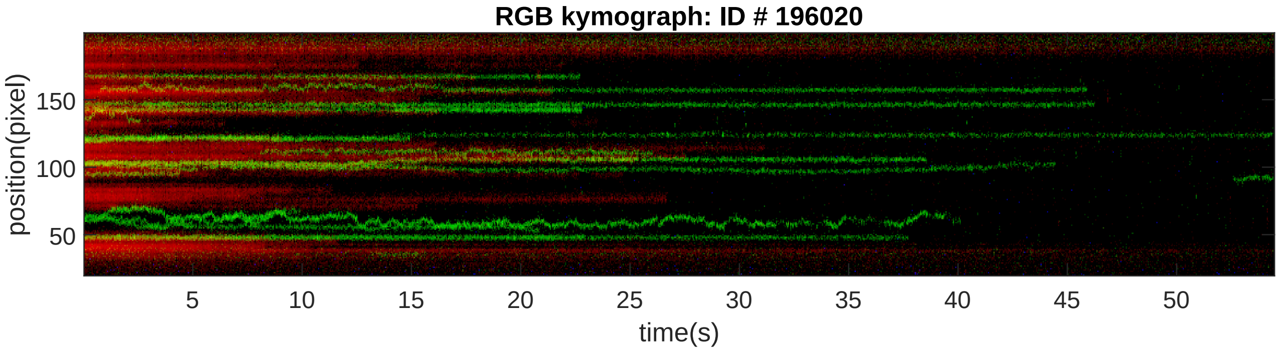
RGB kymograph: ID # 196020
position(pixel)
time(s)
50
100
150
5
10
15
20
25
30
35
40
45
50

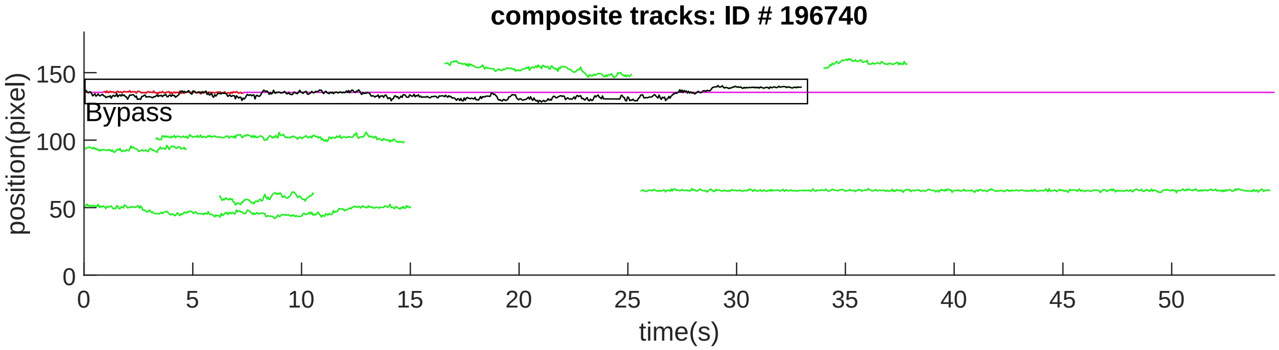
composite tracks: ID # 196740
position(pixel)
Bypass
time(s)
0
5
10
15
20
25
30
35
40
45
50
150
100
50

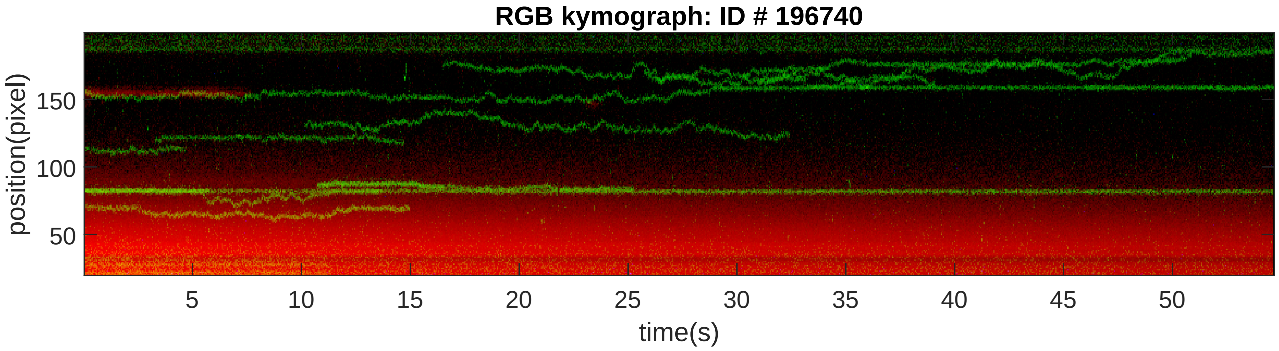
RGB kymograph: ID # 196740
position(pixel)
time(s)
5
10
15
20
25
30
35
40
45
50
150
100
50

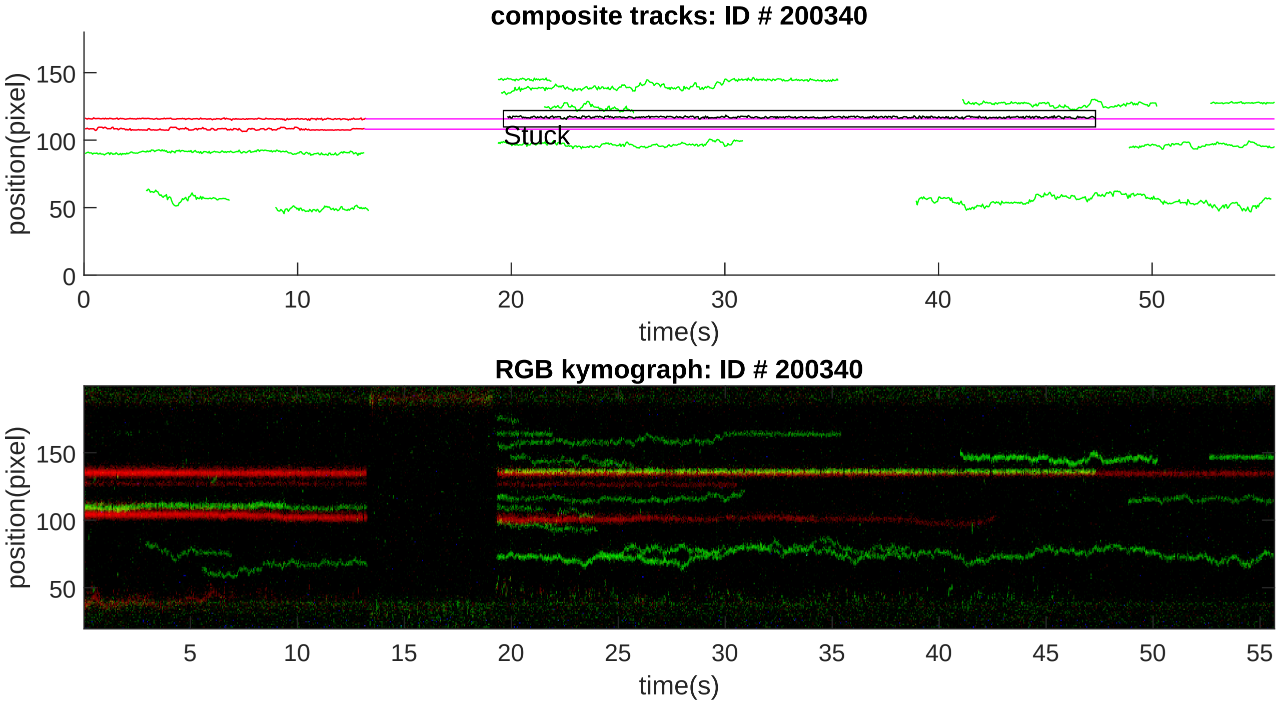
composite tracks: ID # 200340
position(pixel)
Stuck
time(s)
RGB kymograph: ID # 200340
position(pixel)
time(s)

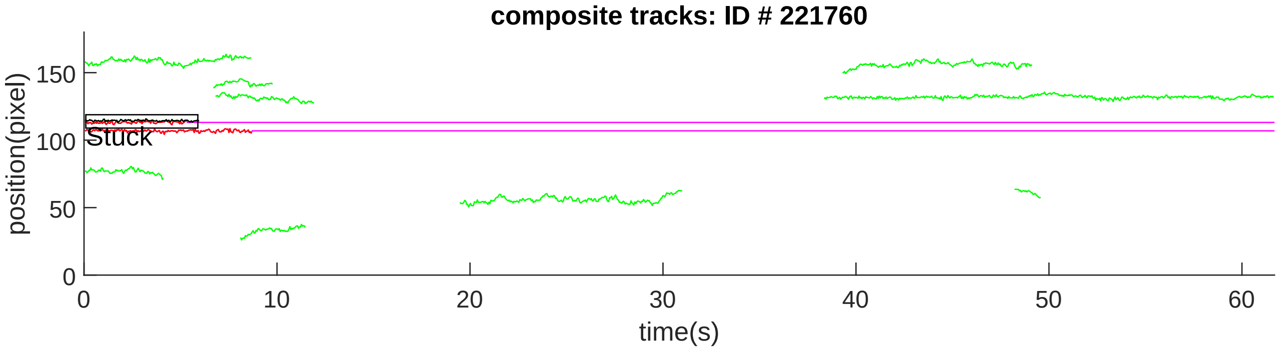
composite tracks: ID # 221760
position(pixel)
time(s)
Stuck
0
50
100
150
0
10
20
30
40
50
60

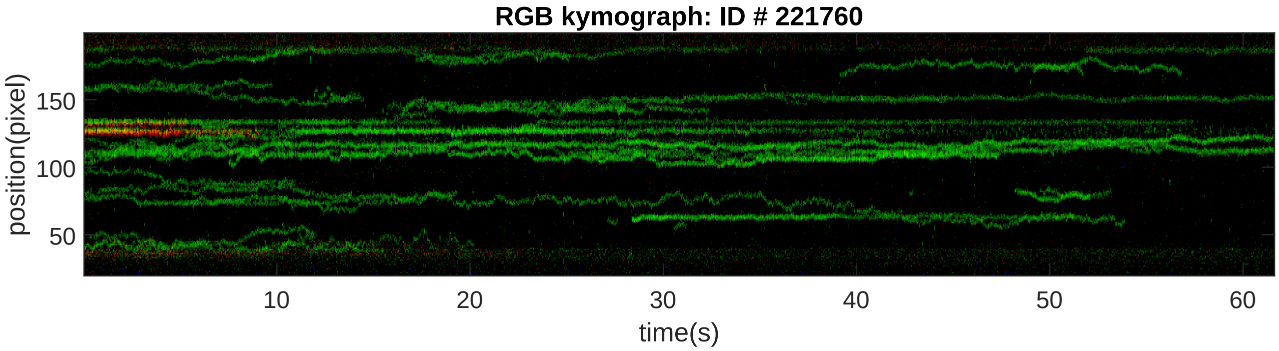
RGB kymograph: ID # 221760
position(pixel)
time(s)
50
100
150
10
20
30
40
50
60

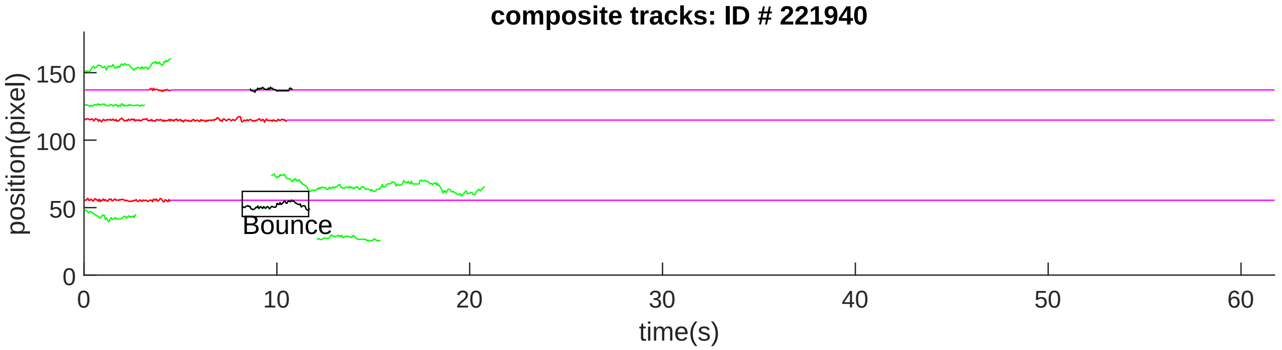
composite tracks: ID # 221940
position(pixel)
time(s)
Bounce
0
50
100
150
10
20
30
40
50
60

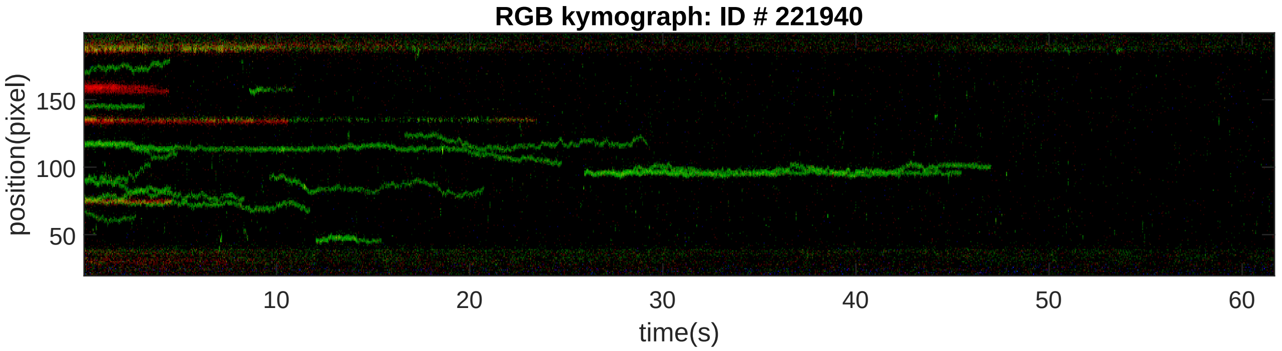
RGB kymograph: ID # 221940
position(pixel)
time(s)
50
100
150
10
20
30
40
50
60

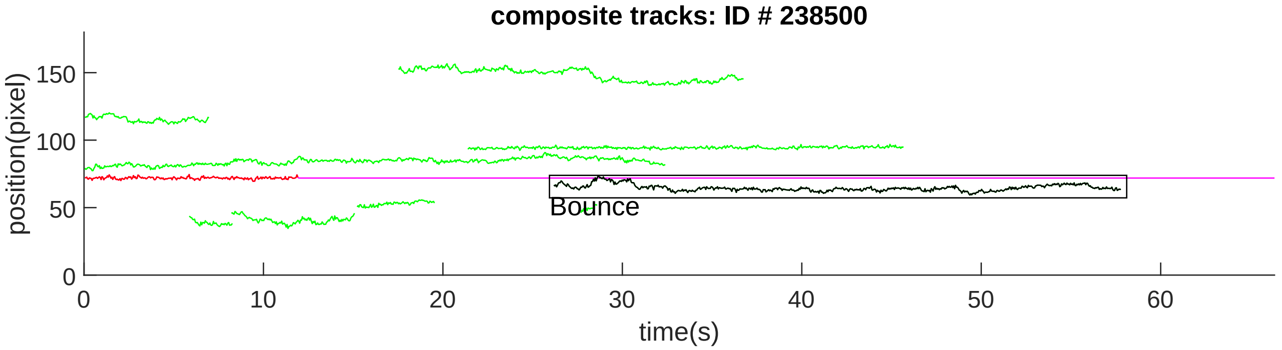
composite tracks: ID # 238500
position(pixel)
time(s)
Bounce
0
50
100
150
0
10
20
30
40
50
60

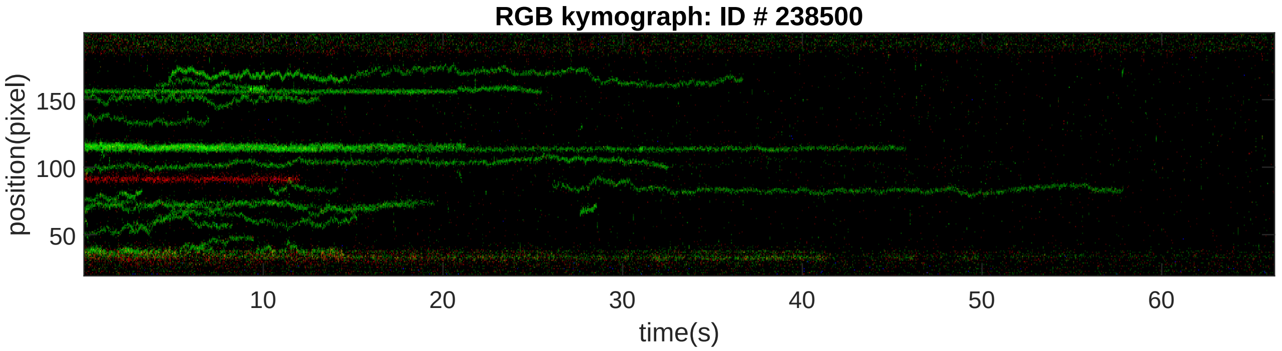
RGB kymograph: ID # 238500
position(pixel)
time(s)
50
100
150
10
20
30
40
50
60

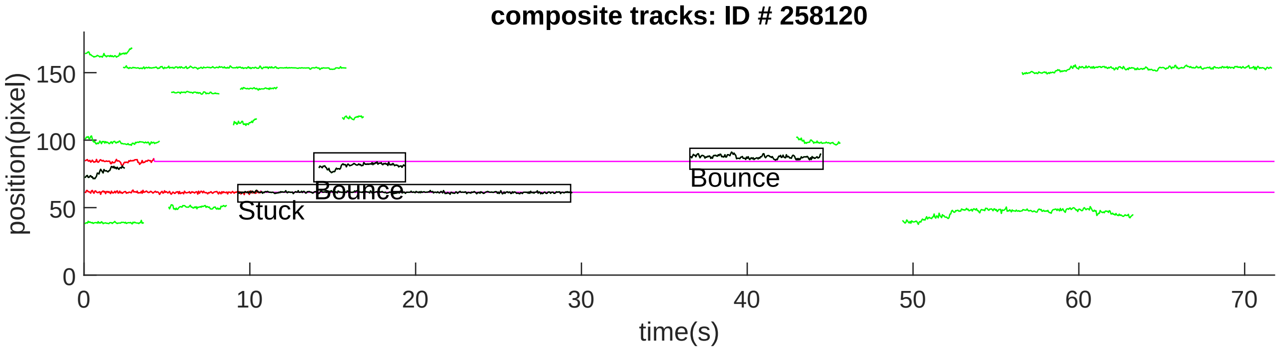
composite tracks: ID # 258120
position(pixel)
time(s)
Bounce
Bounce
Stuck
0
50
100
150
0
10
20
30
40
50
60
70

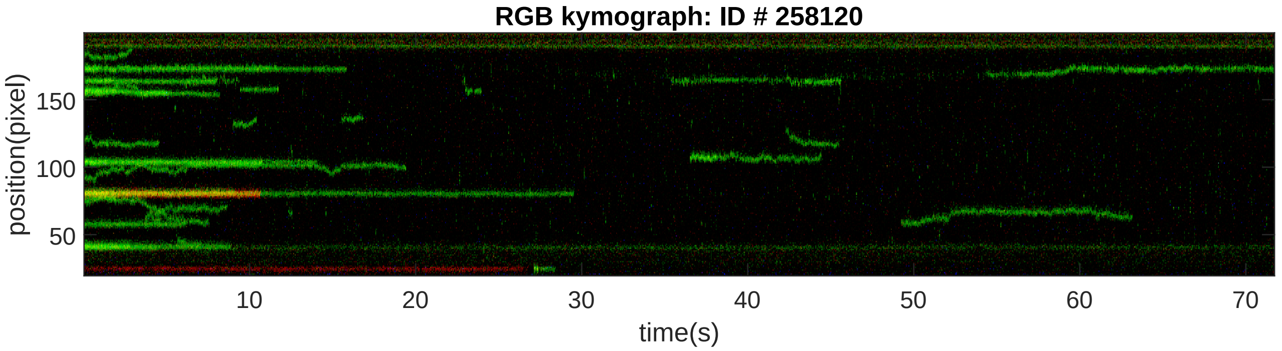
RGB kymograph: ID # 258120
position(pixel)
time(s)
50
100
150
10
20
30
40
50
60
70

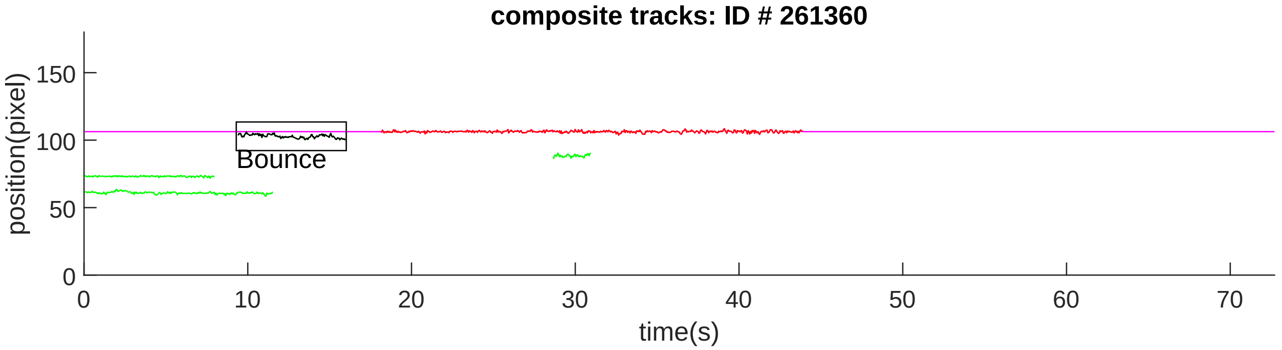
composite tracks: ID # 261360
position(pixel)
Bounce
time(s)
0
10
20
30
40
50
60
70
0
50
100
150

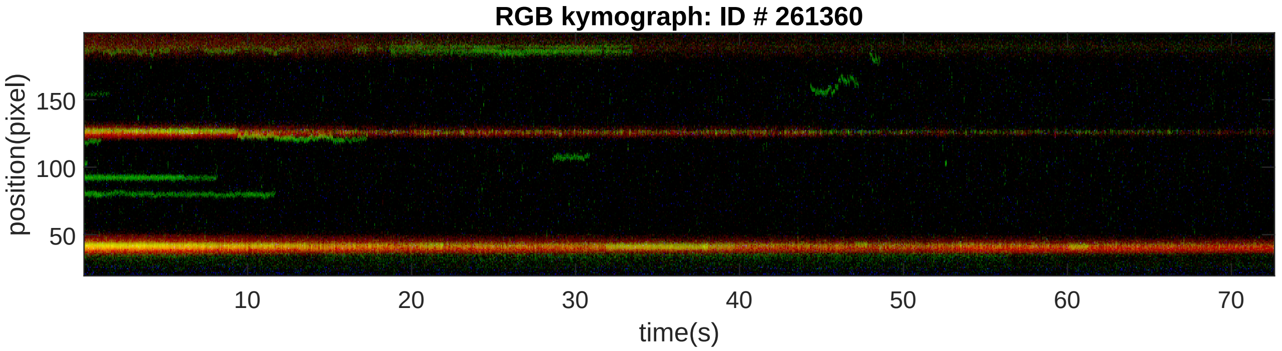
RGB kymograph: ID # 261360
position(pixel)
time(s)
10
20
30
40
50
60
70
50
100
150

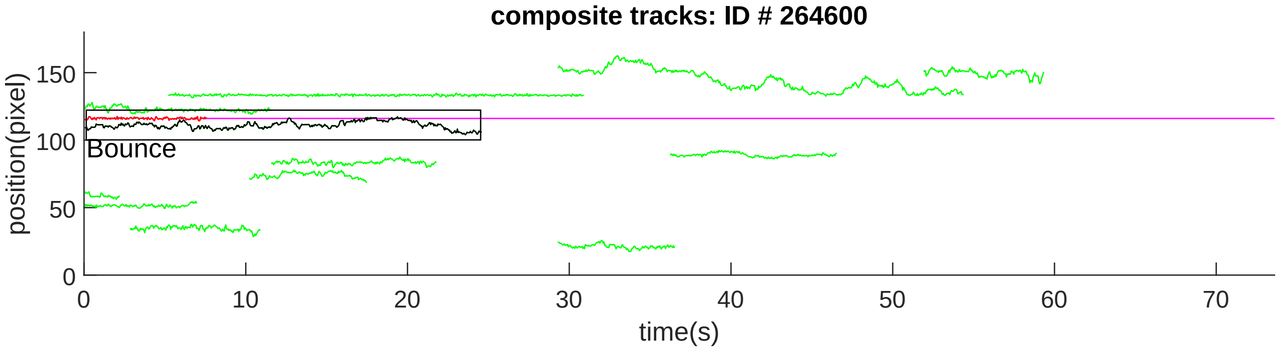
composite tracks: ID # 264600
position(pixel)
time(s)
Bounce
0
50
100
150
0
10
20
30
40
50
60
70

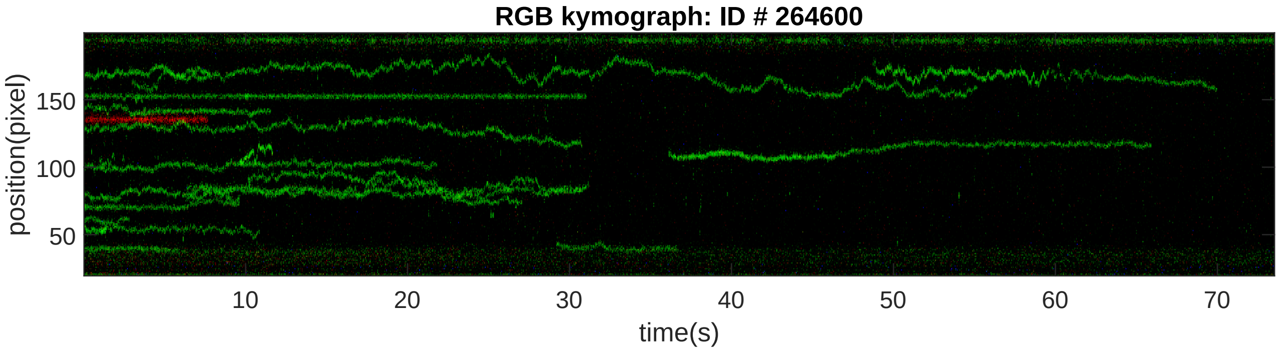
RGB kymograph: ID # 264600
position(pixel)
time(s)
50
100
150
10
20
30
40
50
60
70

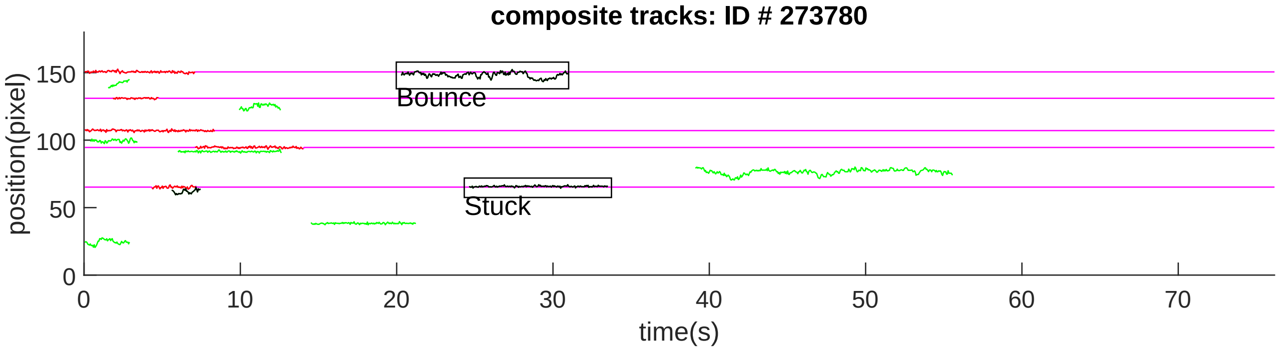

composite tracks: ID # 273780
position(pixel)
150
100
50
0
Bounce
Stuck
0
10
20
30
40
50
60
70
time(s)

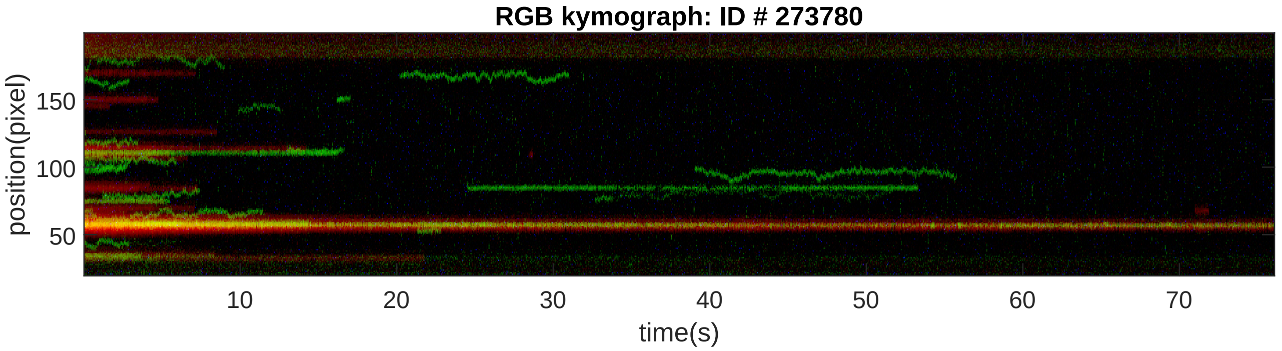

RGB kymograph: ID # 273780
position(pixel)
150
100
50
10
20
30
40
50
60
70
time(s)

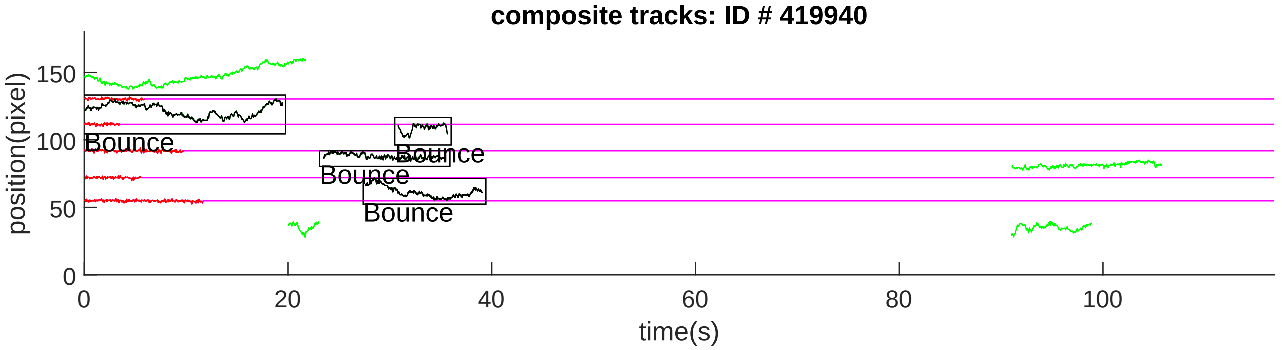
composite tracks: ID # 419940
position(pixel)
time(s)
Bounce
Bounce
Bounce
Bounce
0
50
100
150
0
20
40
60
80
100

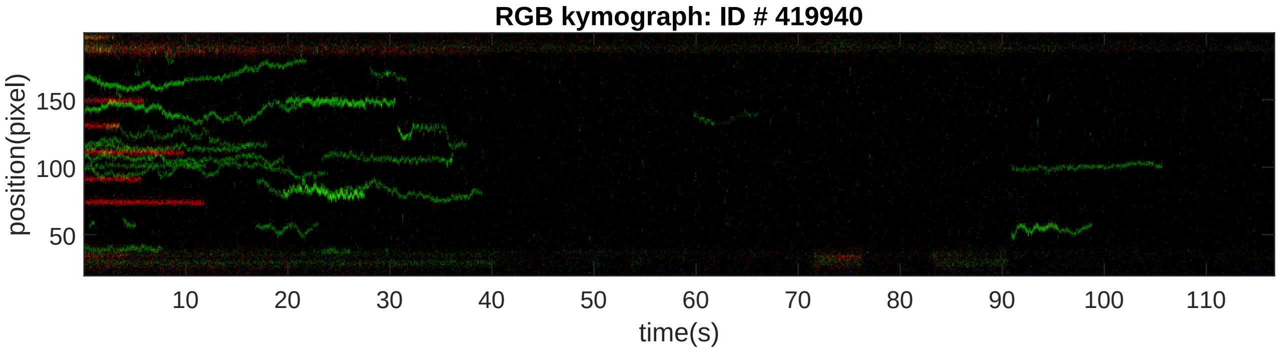
RGB kymograph: ID # 419940
position(pixel)
time(s)
50
100
150
10
20
30
40
50
60
70
80
90
100
110

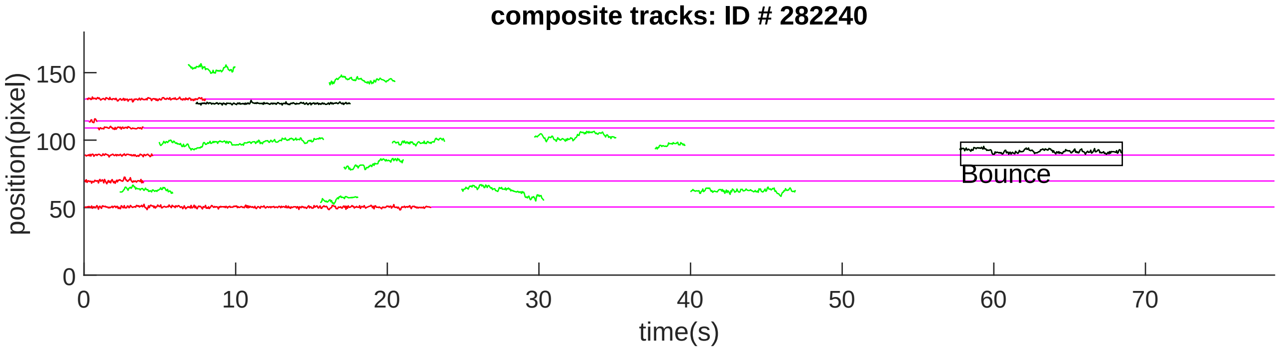

composite tracks: ID # 282240
position(pixel)
150
100
50
0
Bounce
0
10
20
30
40
50
60
70
time(s)

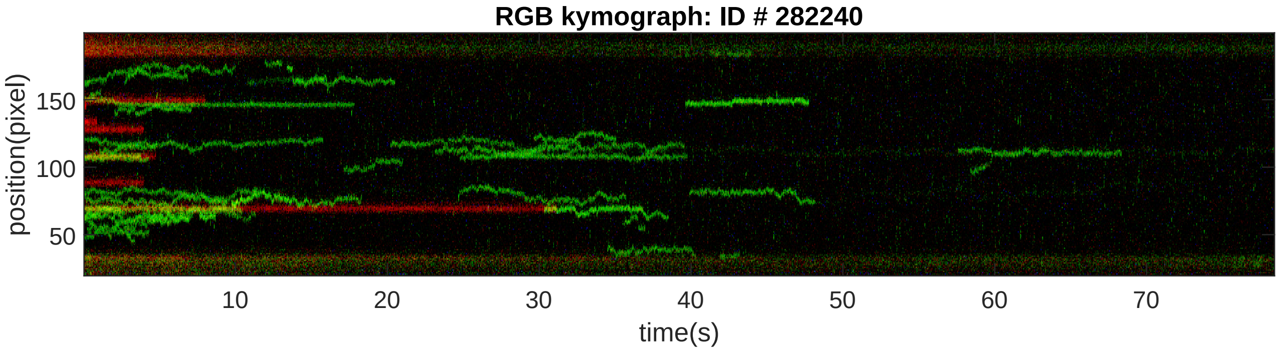

RGB kymograph: ID # 282240
position(pixel)
150
100
50
10
20
30
40
50
60
70
time(s)

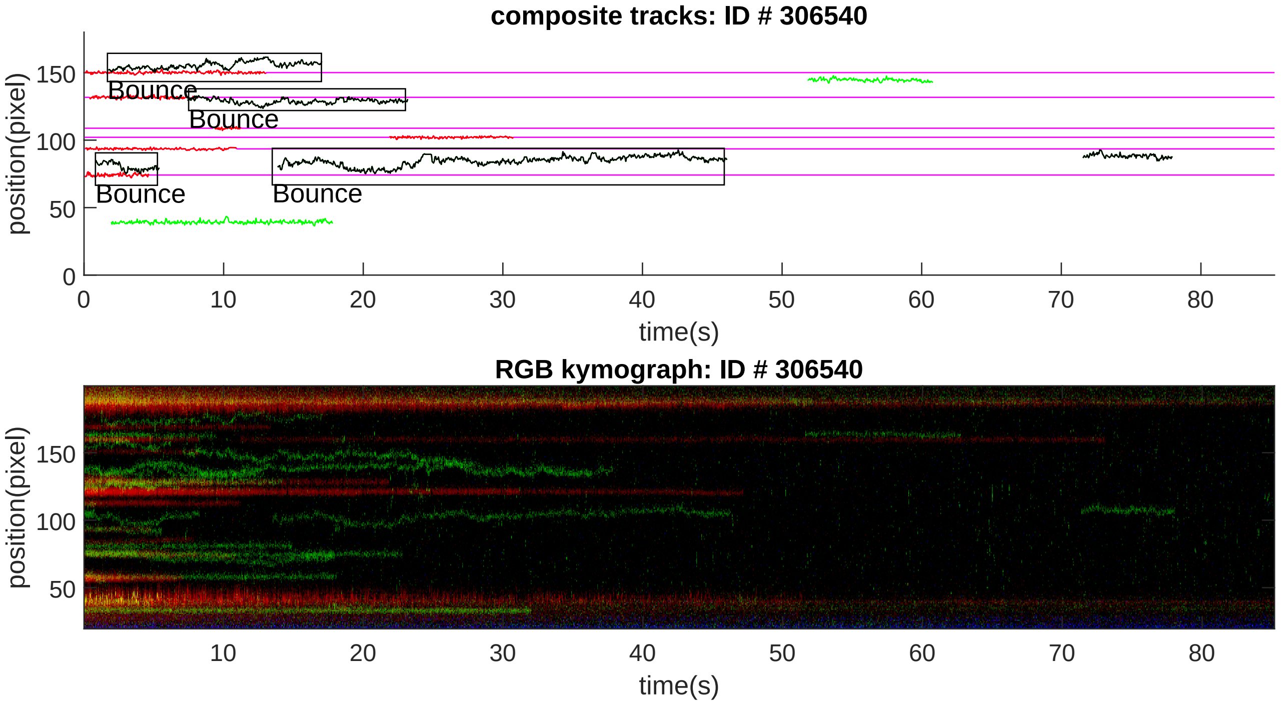
composite tracks: ID # 306540
position(pixel)
time(s)
Bounce
Bounce
Bounce
Bounce
0
50
100
150
0
10
20
30
40
50
60
70
80
RGB kymograph: ID # 306540
position(pixel)
time(s)
50
100
150
10
20
30
40
50
60
70
80

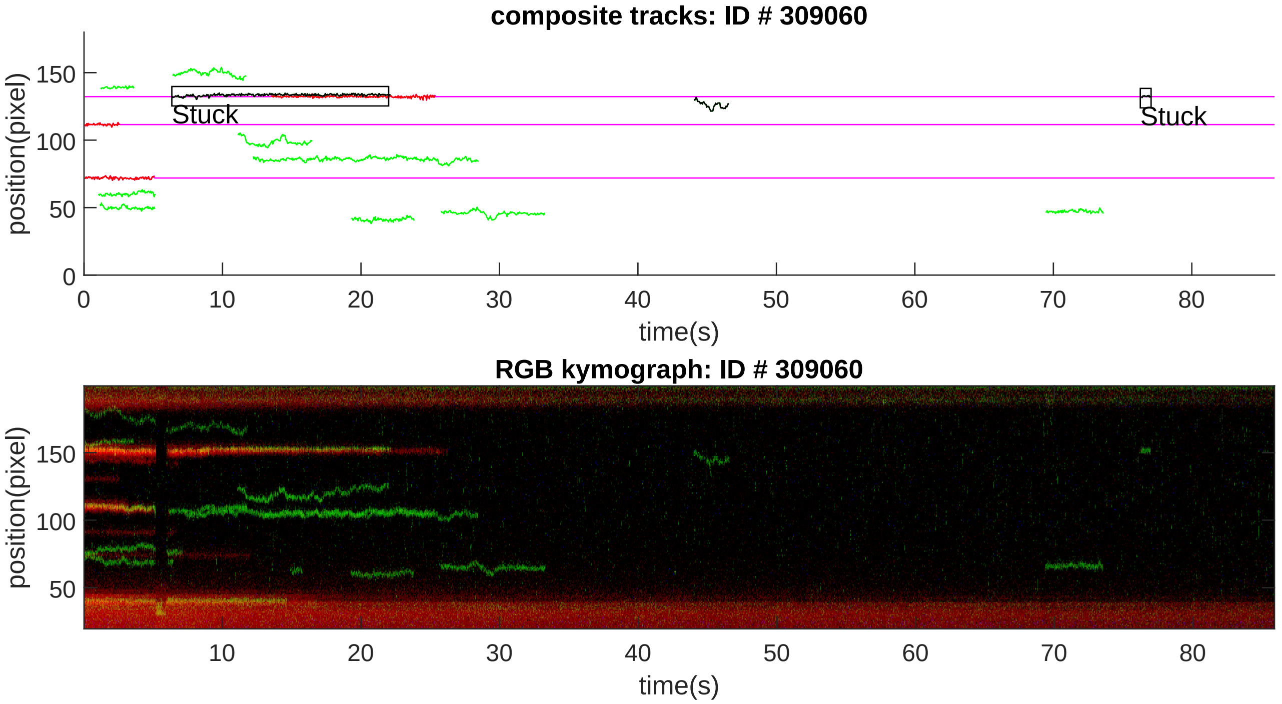
composite tracks: ID # 309060
Stuck
Stuck
position(pixel)
time(s)
0
50
100
150
0
10
20
30
40
50
60
70
80
RGB kymograph: ID # 309060
position(pixel)
time(s)
50
100
150
10
20
30
40
50
60
70
80

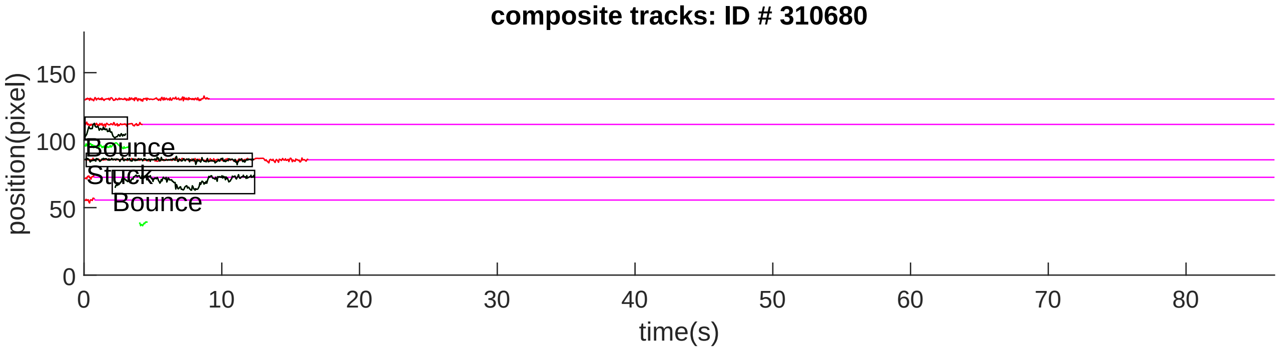
composite tracks: ID # 310680
position(pixel)
time(s)
Bounce
Stuck
Bounce
0
50
100
150
0
10
20
30
40
50
60
70
80

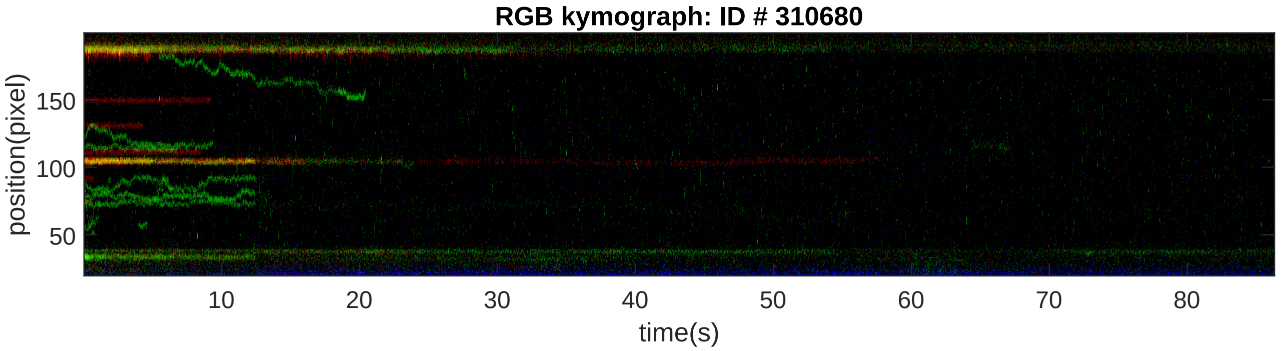
RGB kymograph: ID # 310680
position(pixel)
time(s)
50
100
150
10
20
30
40
50
60
70
80

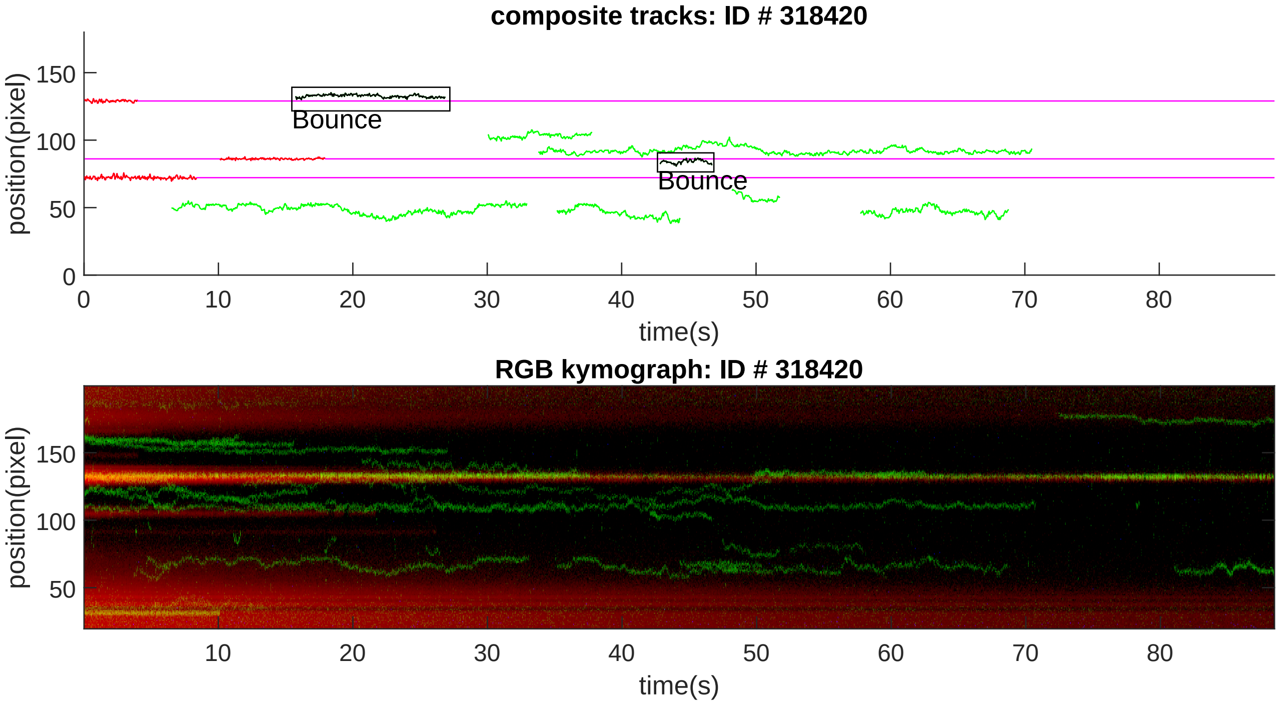

composite tracks: ID # 318420
position(pixel)
time(s)
Bounce
Bounce
RGB kymograph: ID # 318420
position(pixel)
time(s)

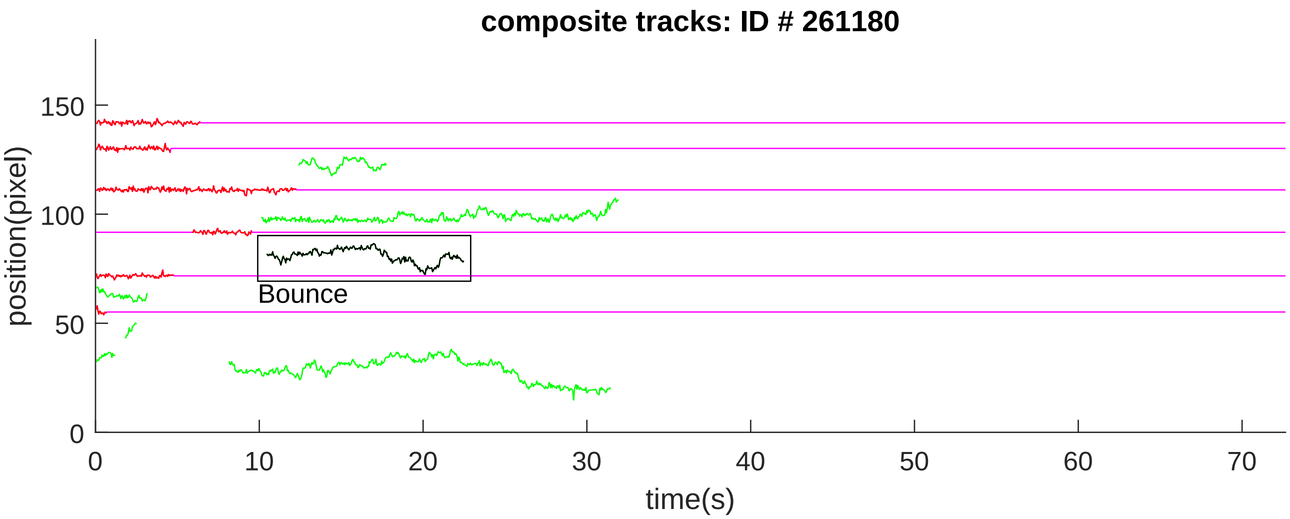
composite tracks: ID # 261180
position(pixel)
time(s)
Bounce
0
50
100
150
0
10
20
30
40
50
60
70

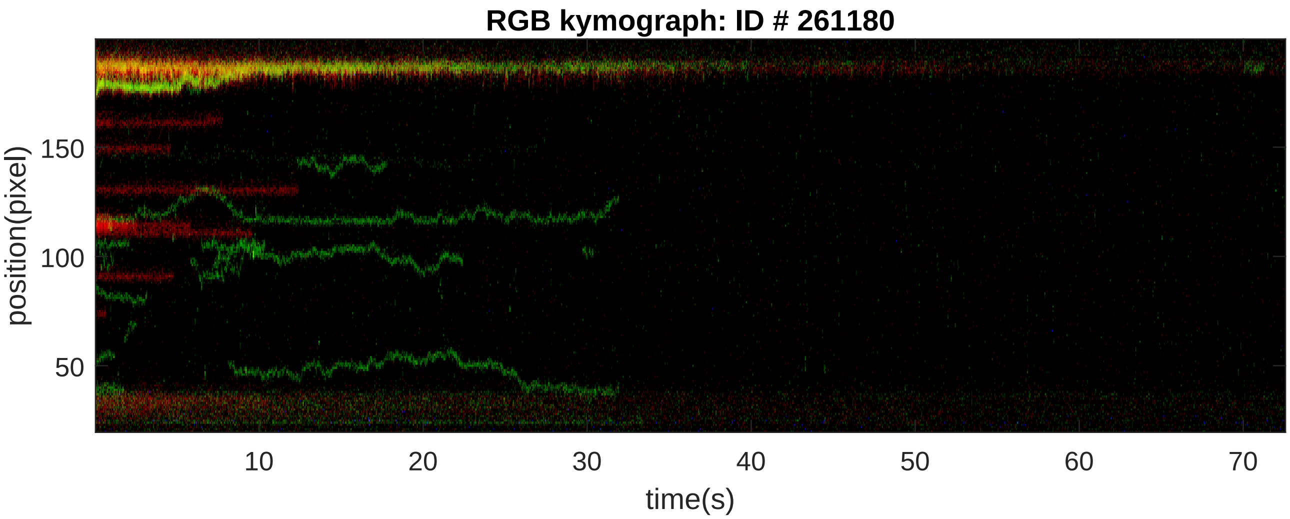
RGB kymograph: ID # 261180
position(pixel)
time(s)
50
100
150
10
20
30
40
50
60
70

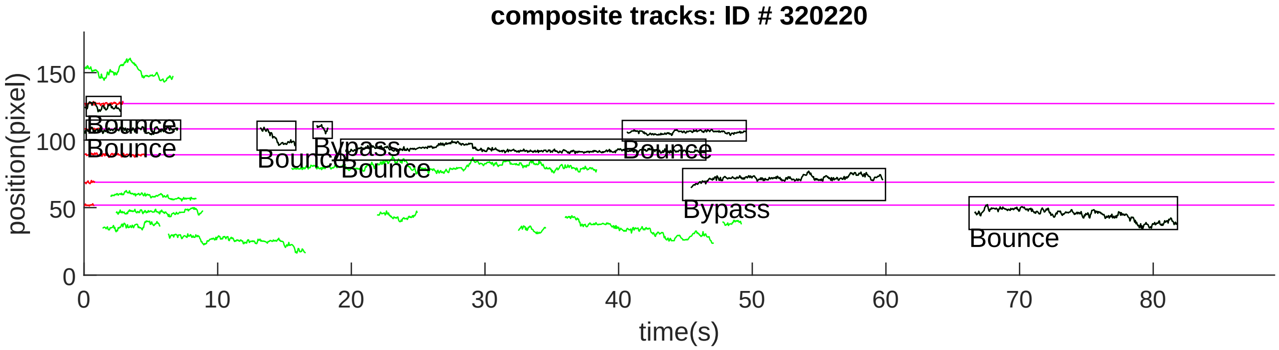
composite tracks: ID # 320220
position(pixel)
time(s)
Bounce
Bounce
Bounce
Bypass
Bounce
Bounce
Bypass
Bounce
0
50
100
150
0
10
20
30
40
50
60
70
80

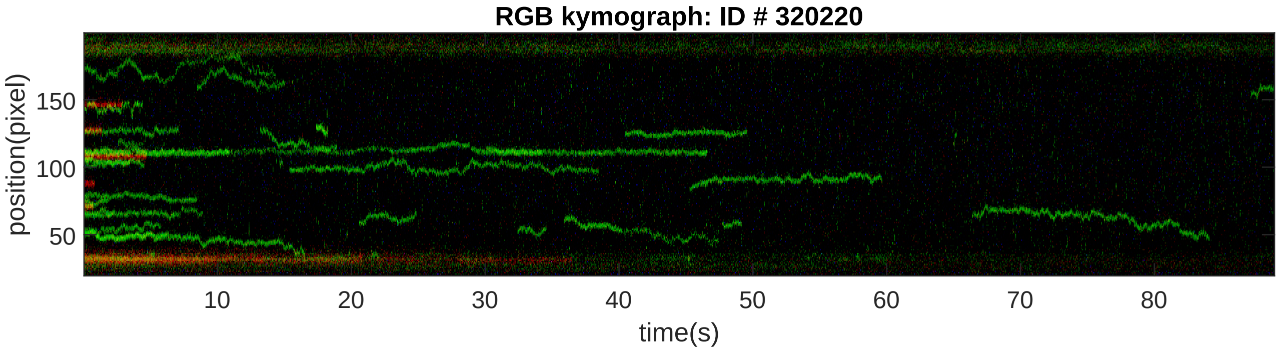
RGB kymograph: ID # 320220
position(pixel)
time(s)
50
100
150
10
20
30
40
50
60
70
80

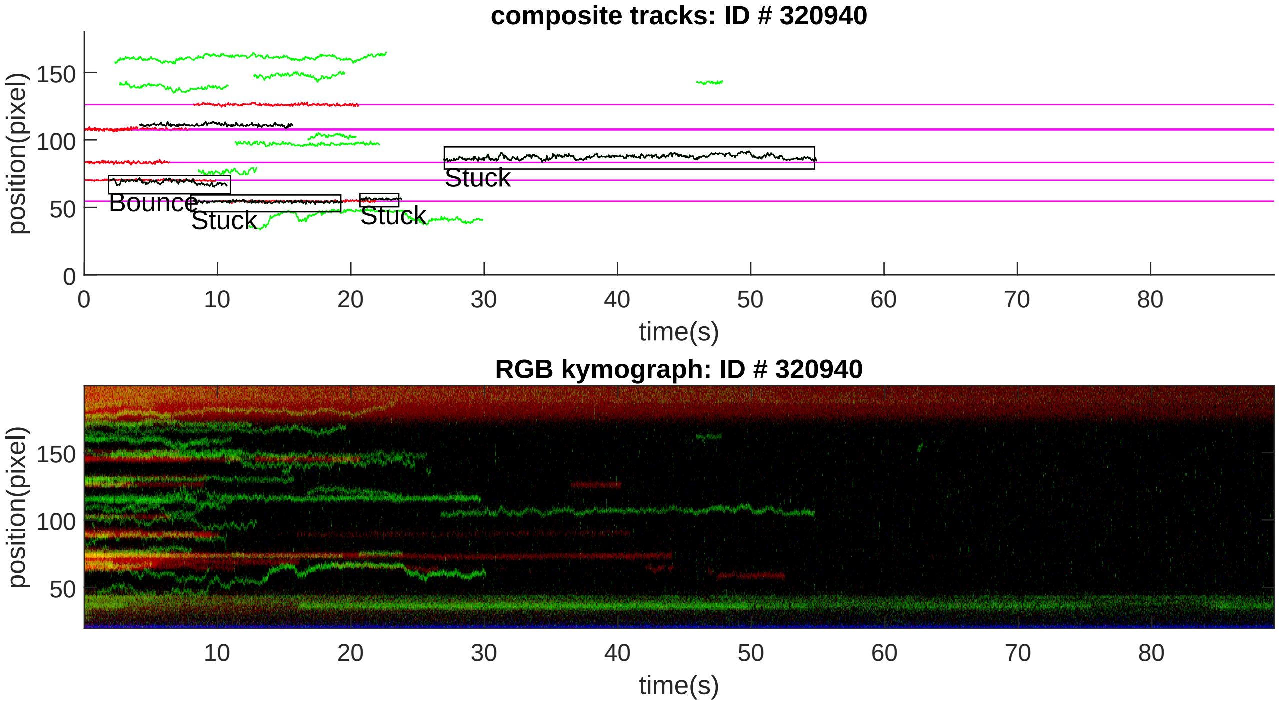
composite tracks: ID # 320940
position(pixel)
time(s)
Bounce
Stuck
Stuck
Stuck
RGB kymograph: ID # 320940
position(pixel)
time(s)

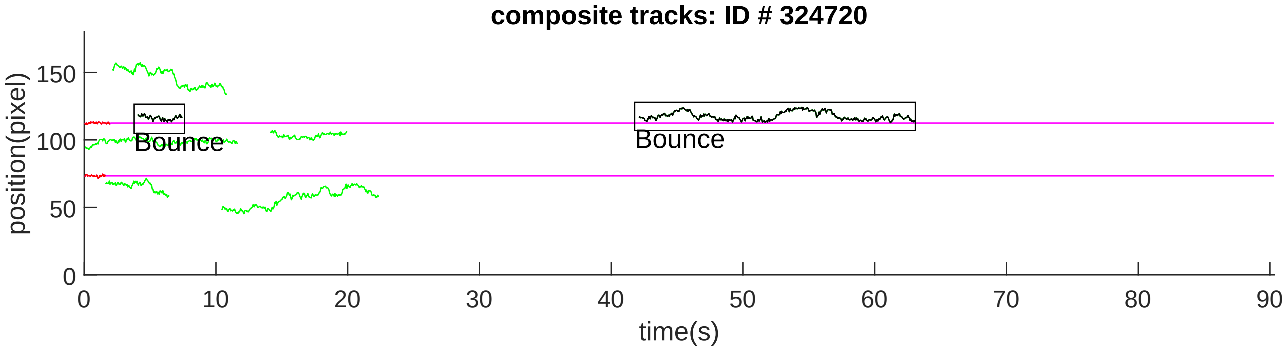

composite tracks: ID # 324720
position(pixel)
Bounce
Bounce
time(s)

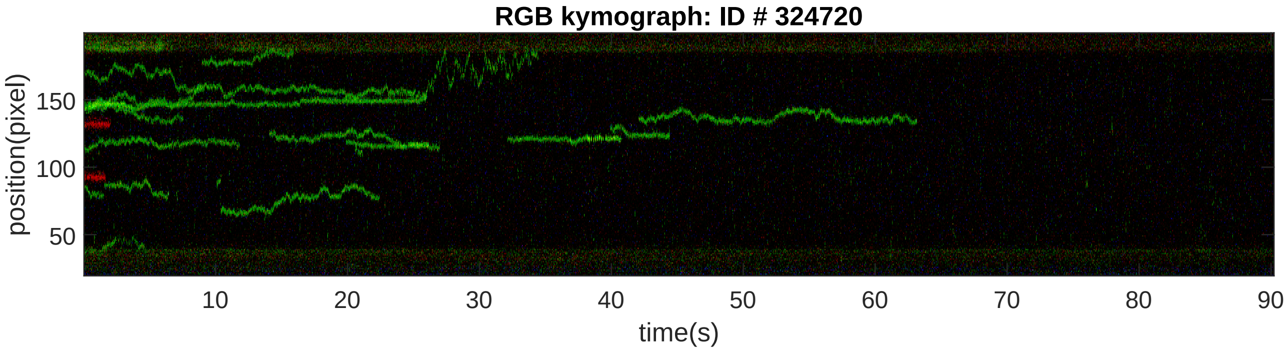

RGB kymograph: ID # 324720
position(pixel)
time(s)

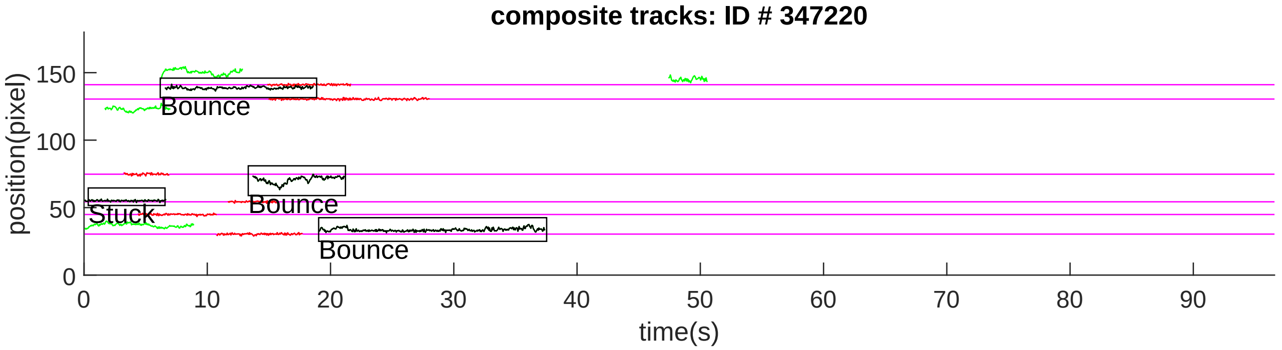
composite tracks: ID # 347220
position(pixel)
150
100
50
0
Bounce
Stuck
Bounce
Bounce
0
10
20
30
40
50
60
70
80
90
time(s)

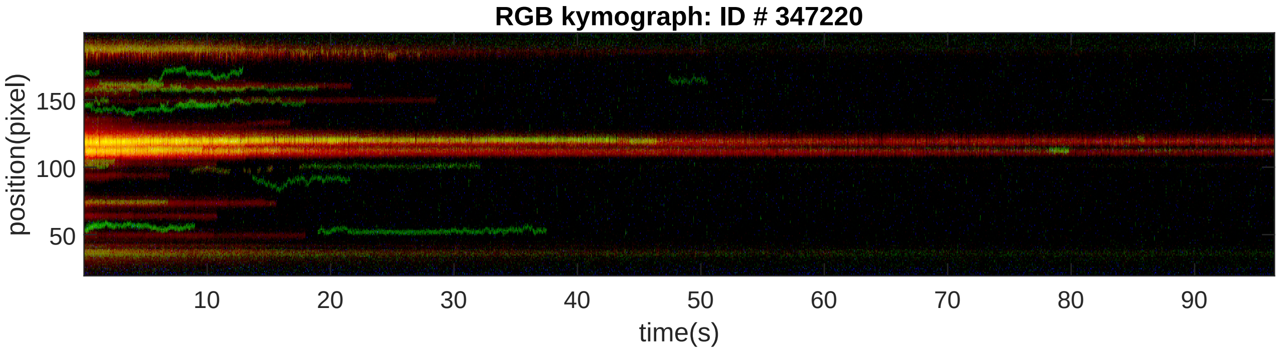
RGB kymograph: ID # 347220
position(pixel)
150
100
50
10
20
30
40
50
60
70
80
90
time(s)

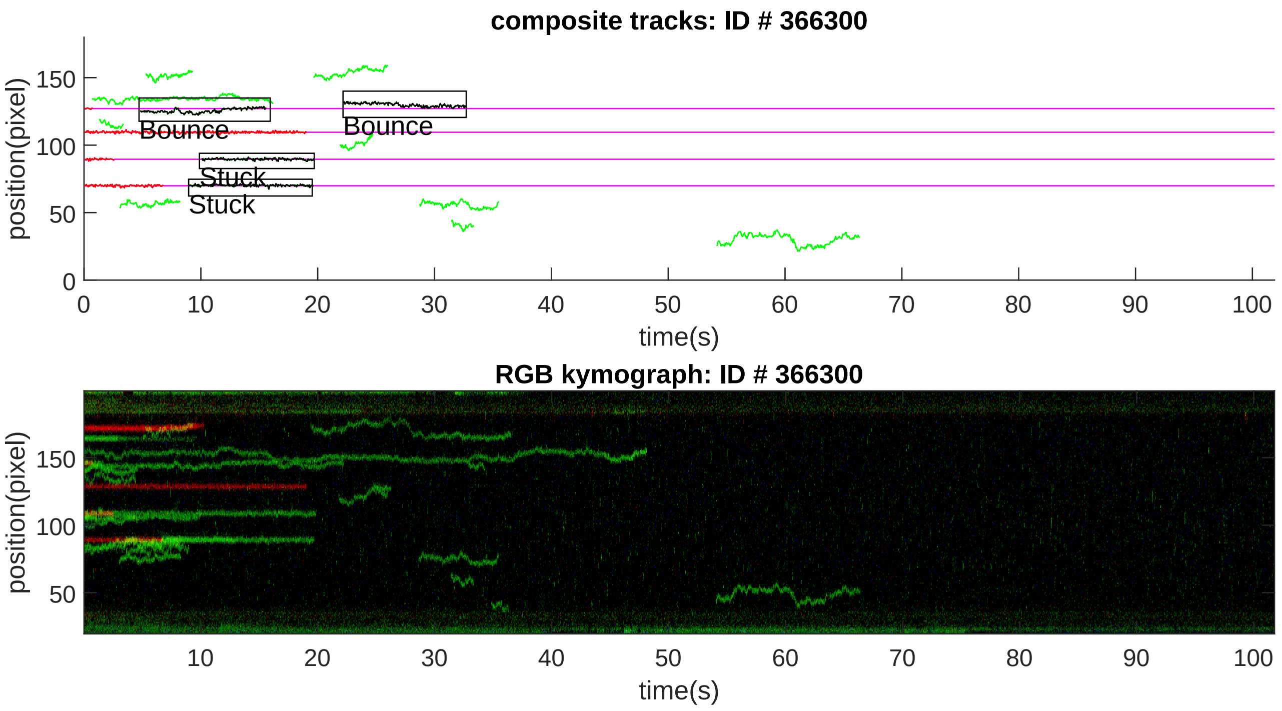
composite tracks: ID # 366300
position(pixel)
time(s)
Bounce
Bounce
Stuck
Stuck
RGB kymograph: ID # 366300
position(pixel)
time(s)

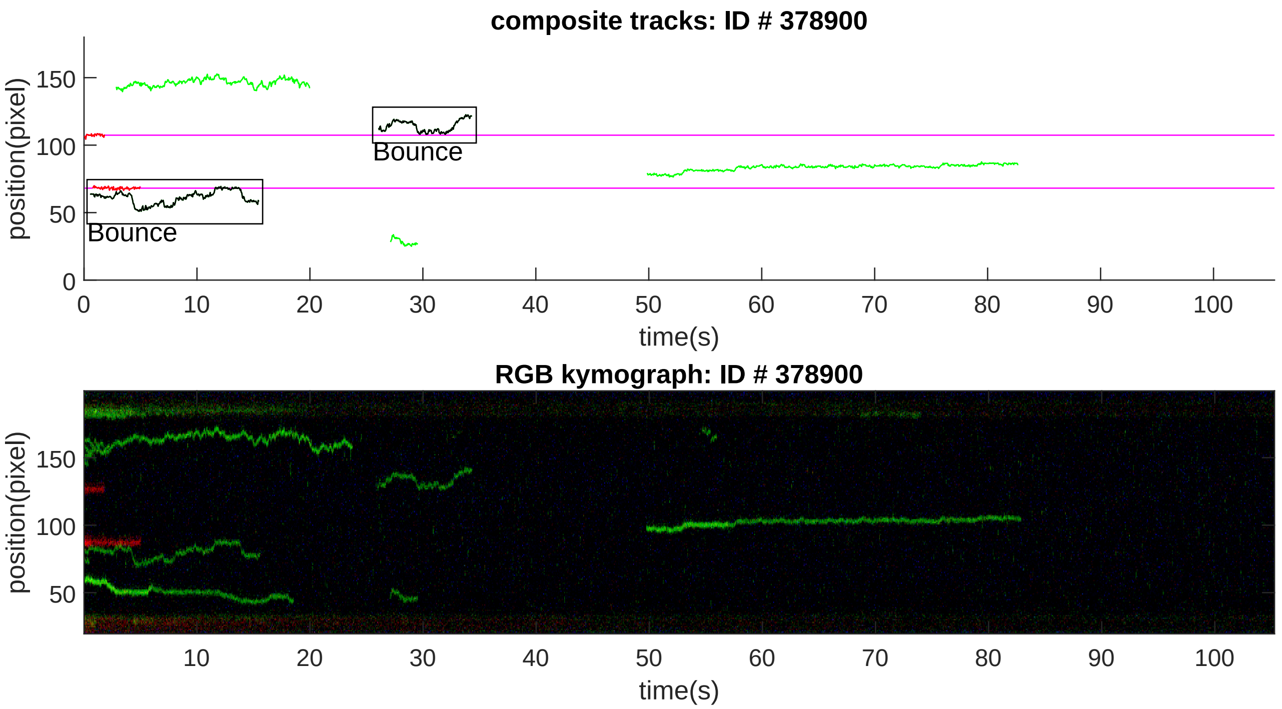

composite tracks: ID # 378900
position(pixel)
time(s)
Bounce
Bounce
RGB kymograph: ID # 378900
position(pixel)
time(s)

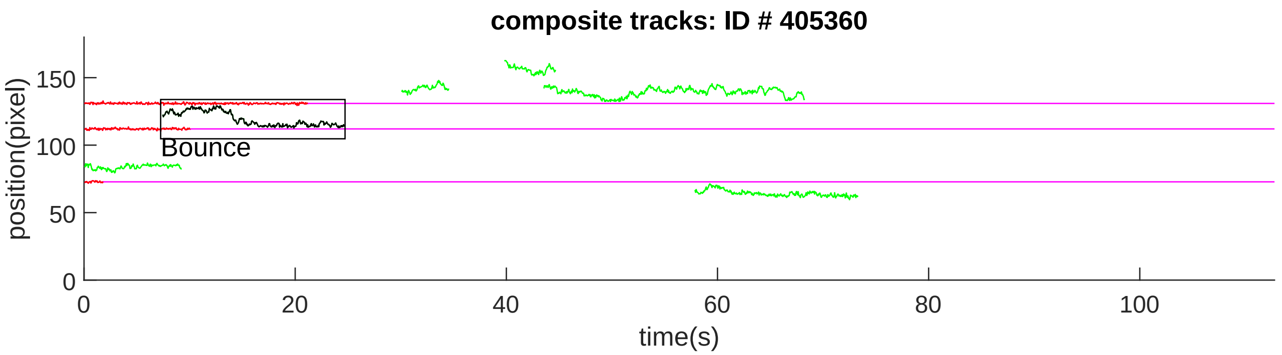
composite tracks: ID # 405360
position(pixel)
time(s)
Bounce
0
50
100
150
0
20
40
60
80
100

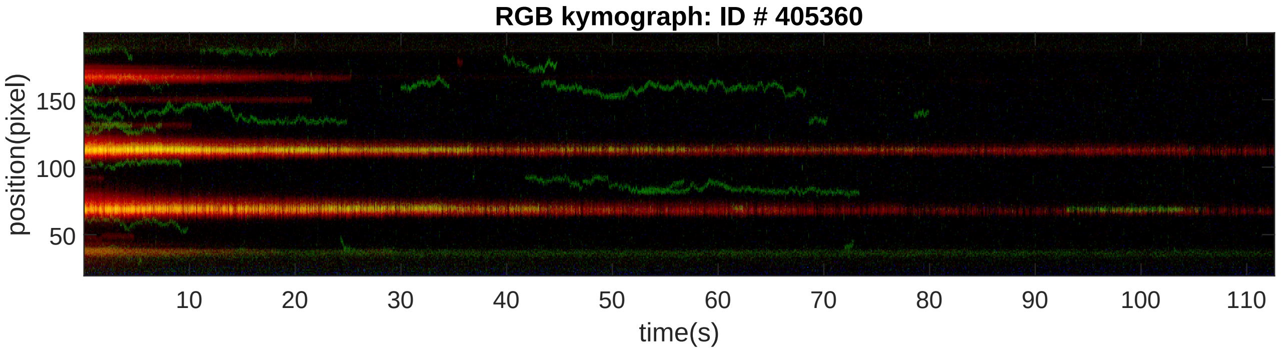
RGB kymograph: ID # 405360
position(pixel)
time(s)
50
100
150
10
20
30
40
50
60
70
80
90
100
110

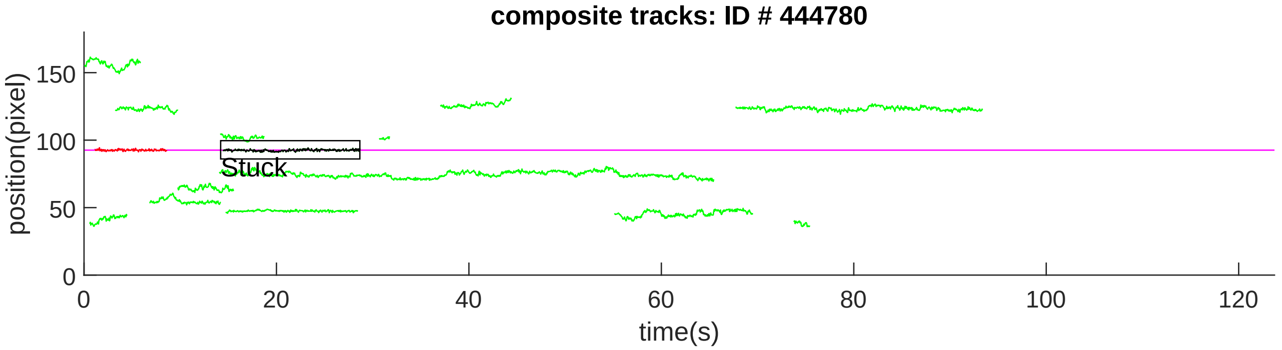
composite tracks: ID # 444780
position(pixel)
Stuck
time(s)

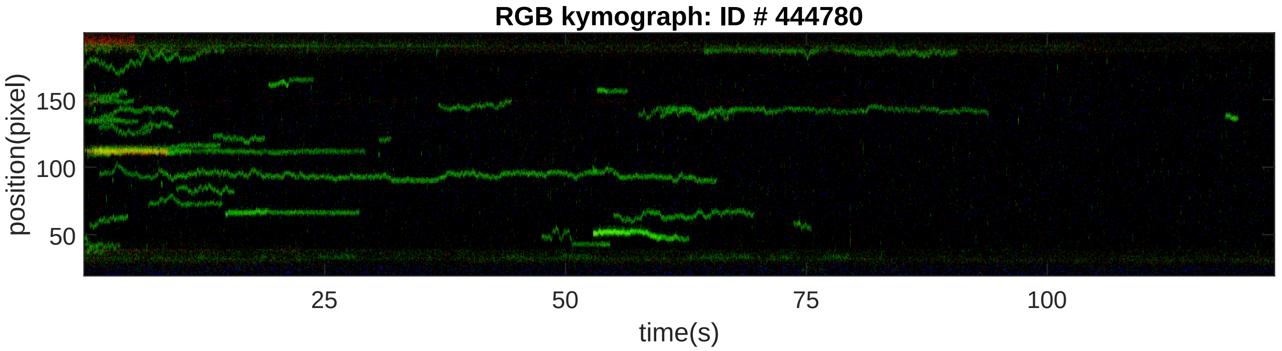
RGB kymograph: ID # 444780
position(pixel)
time(s)

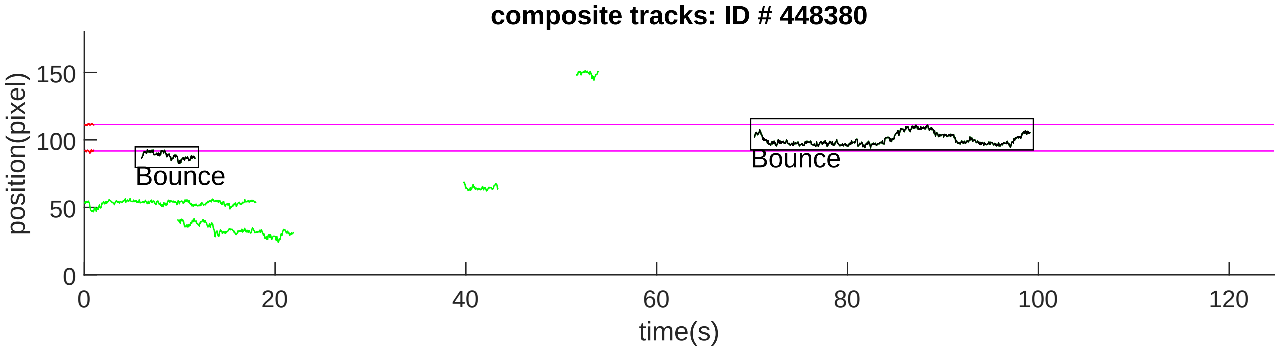
composite tracks: ID # 448380
position(pixel)
time(s)
Bounce
Bounce
0
50
100
150
0
20
40
60
80
100
120

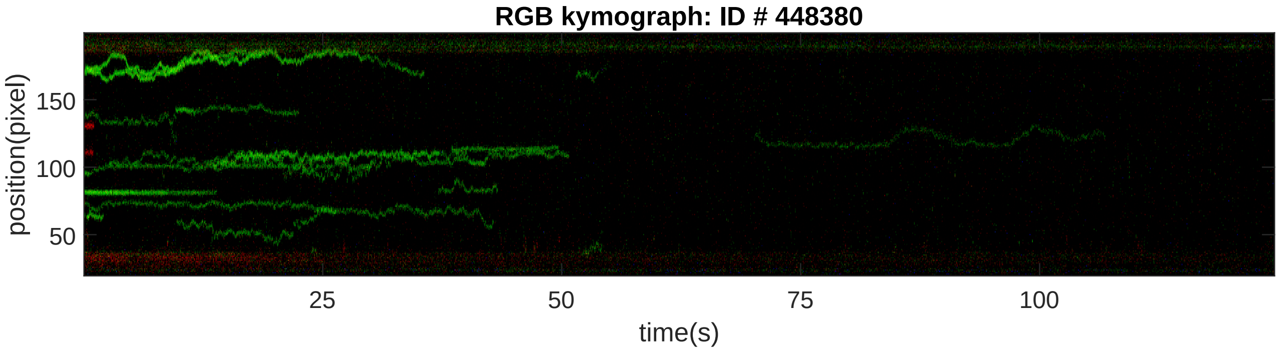
RGB kymograph: ID # 448380
position(pixel)
time(s)
50
100
150
25
50
75
100

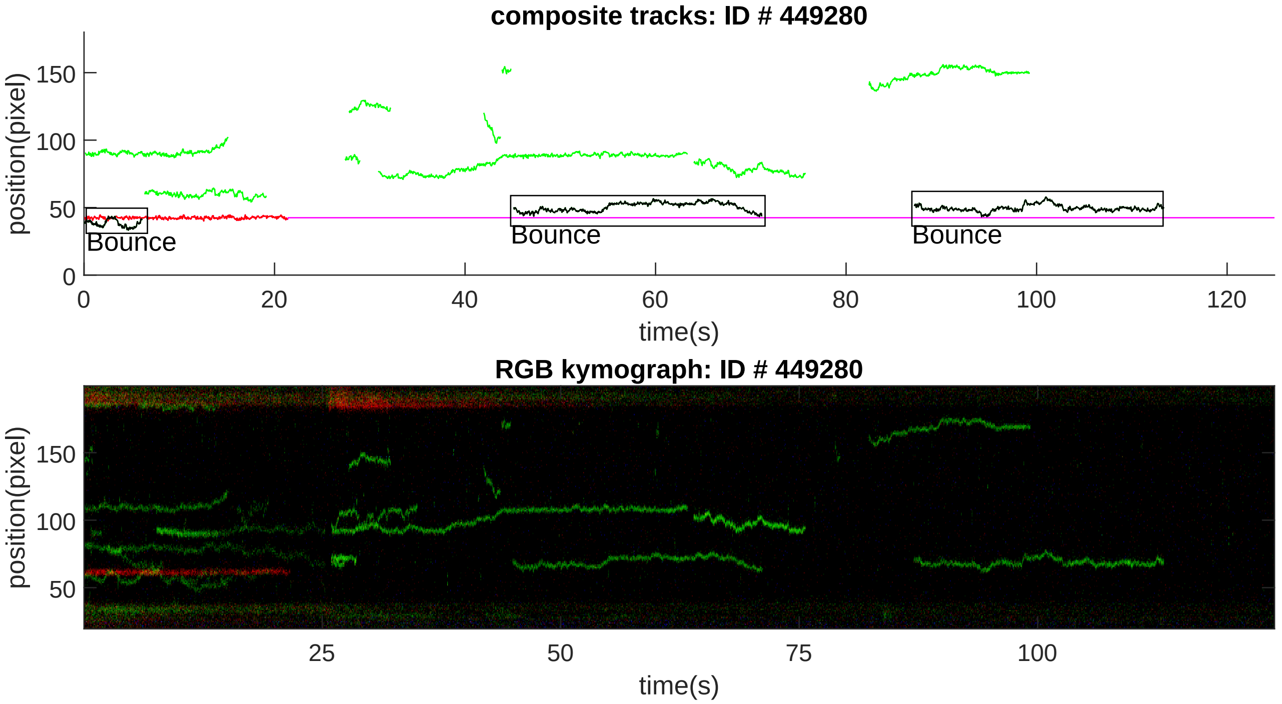
composite tracks: ID # 449280
position(pixel)
time(s)
Bounce
Bounce
Bounce
RGB kymograph: ID # 449280
position(pixel)
time(s)

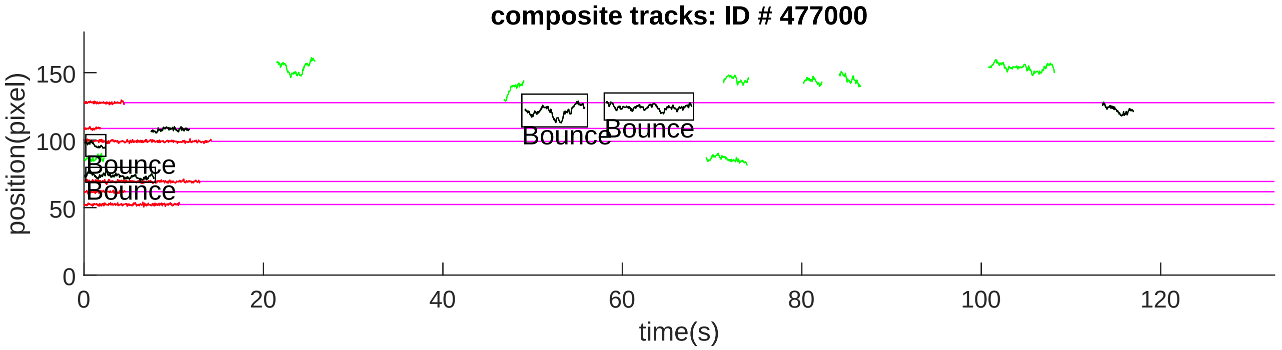
composite tracks: ID # 477000
position(pixel)
time(s)
Bounce
Bounce
Bounce
Bounce
0
50
100
150
0
20
40
60
80
100
120

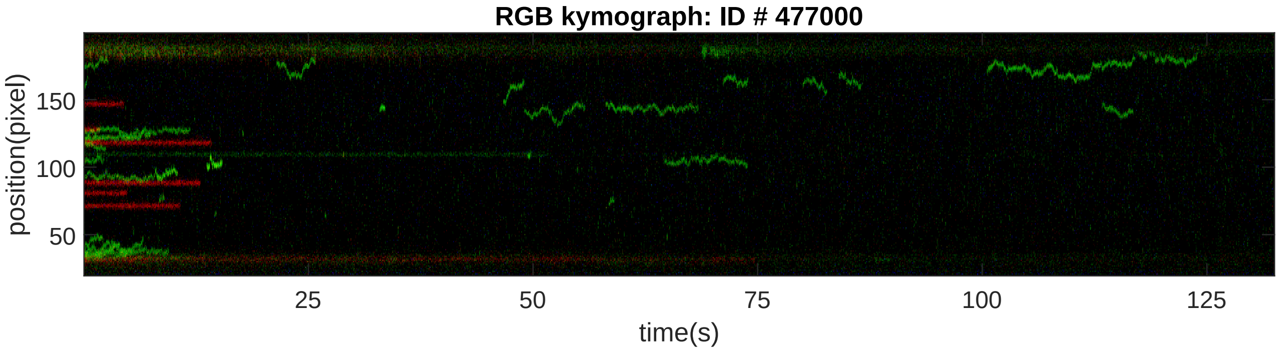
RGB kymograph: ID # 477000
position(pixel)
time(s)
50
100
150
25
50
75
100
125

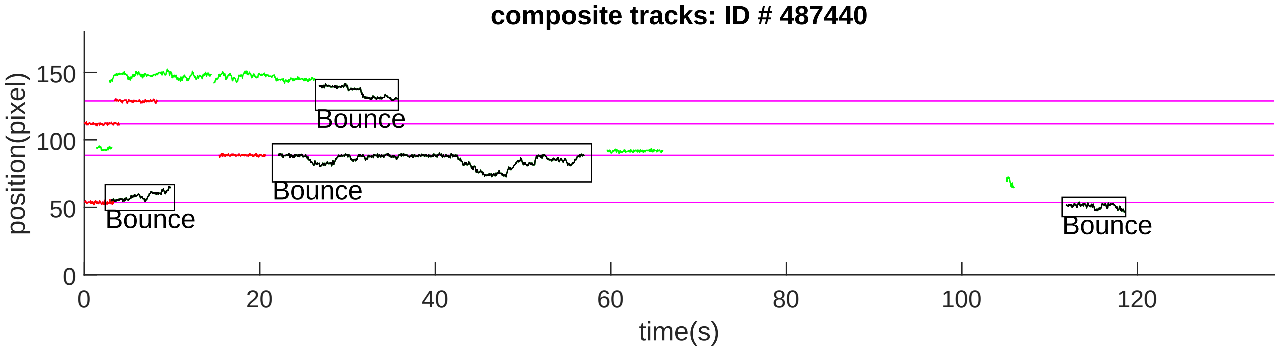
composite tracks: ID # 487440
position(pixel)
150
100
50
0
Bounce
Bounce
Bounce
Bounce
0
20
40
60
80
100
120
time(s)

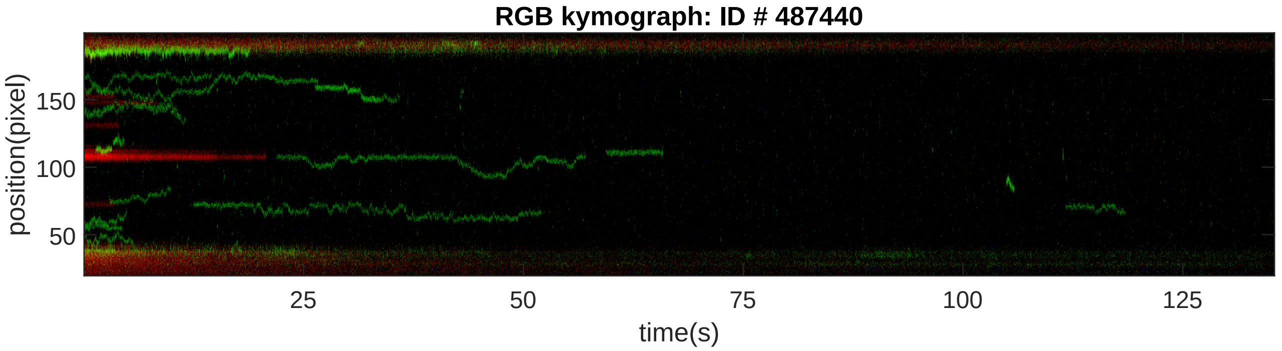
RGB kymograph: ID # 487440
position(pixel)
150
100
50
25
50
75
100
125
time(s)

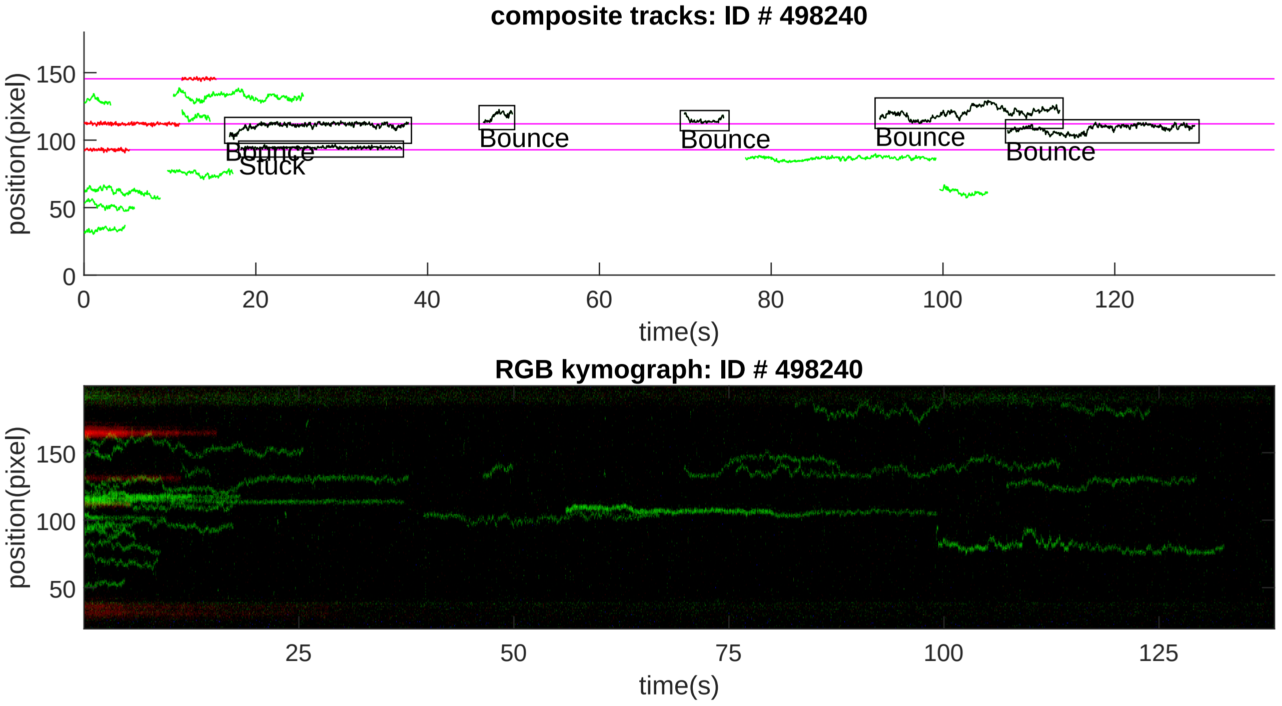
composite tracks: ID # 498240
position(pixel)
0
50
100
150
0
20
40
60
80
100
120
time(s)
Bounce
Stuck
Bounce
Bounce
Bounce
Bounce
RGB kymograph: ID # 498240
position(pixel)
50
100
150
25
50
75
100
125
time(s)

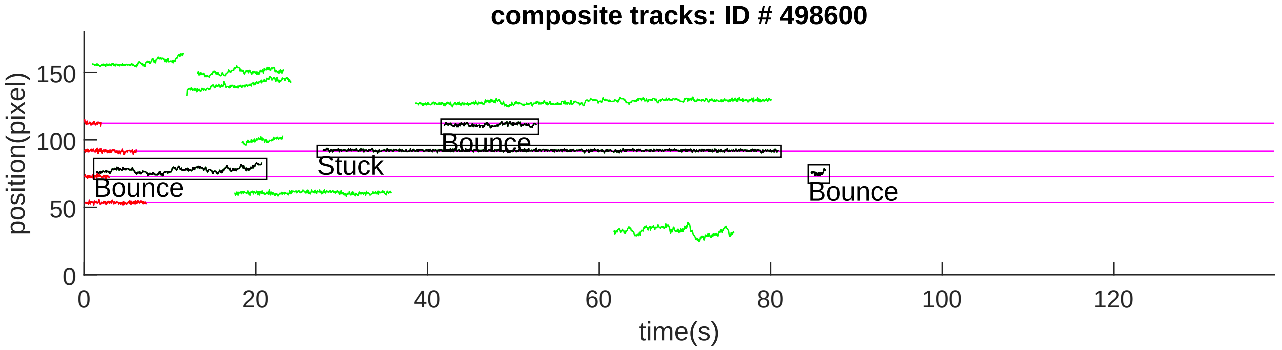
composite tracks: ID # 498600
position(pixel)
150
100
50
0
0
20
40
60
80
100
120
time(s)
Bounce
Stuck
Bounce
Bounce

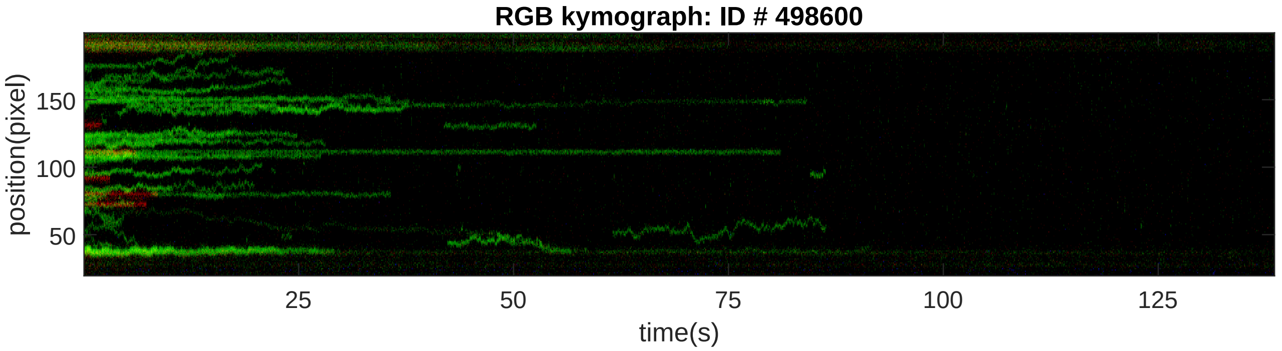
RGB kymograph: ID # 498600
position(pixel)
150
100
50
25
50
75
100
125
time(s)

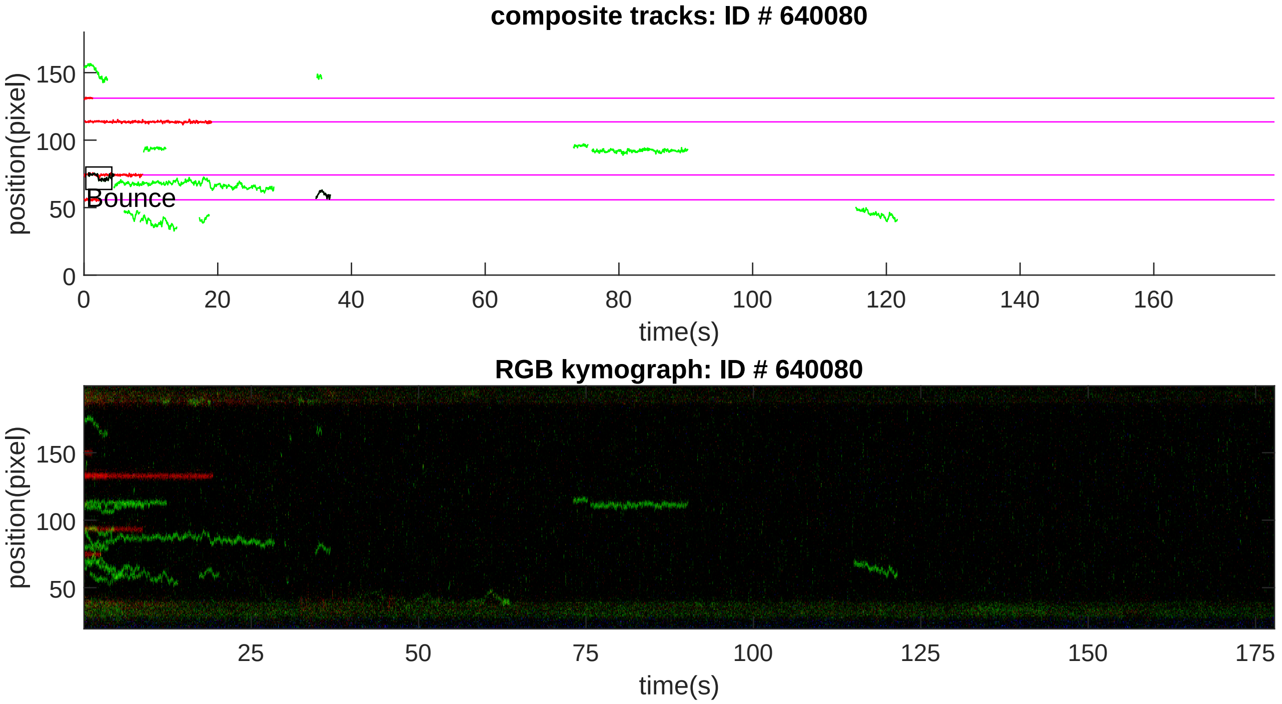
composite tracks: ID # 640080
position(pixel)
150
100
50
0
Bounce
0
20
40
60
80
100
120
140
160
time(s)
RGB kymograph: ID # 640080
position(pixel)
150
100
50
25
50
75
100
125
150
175
time(s)
