# Supplementary material for: ATP binding facilitates target search of SWR1 chromatin remodeler by promoting one-dimensional diffusion on DNA
Source: eLife. 2022 Jul 25;11:e77352. doi: 10.7554/eLife.77352 (PMC9365391; doi:10.7554/eLife.77352)
Supplement: Transparent reporting form [file elife-77352-transrepform1.docx]

***eLife’s* transparent reporting form**

We encourage authors to provide detailed information *within their submission* to facilitate the interpretation and replication of experiments. Authors can upload supporting documentation to indicate the use of appropriate reporting guidelines for health-related research (see [EQUATOR Network](http://www.equator-network.org/%20)), life science research (see the [BioSharing Information Resource](https://biosharing.org/)), or the [ARRIVE guidelines](http://www.plosbiology.org/article/info:doi/10.1371/journal.pbio.1000412) for reporting work involving animal research. Where applicable, authors should refer to any relevant reporting standards documents in this form.

If you have any questions, please consult our Journal Policies and/or contact us: [editorial@elifesciences.org](mailto:editorial@elifesciences.org).

**Sample-size estimation**

- You should state whether an appropriate sample size was computed when the study was being designed
- You should state the statistical method of sample size computation and any required assumptions
- If no explicit power analysis was used, you should describe how you decided what sample (replicate) size (number) to use

Please outline where this information can be found within the submission (e.g., sections or figure legends), or explain why this information doesn’t apply to your submission:

No explicit power analysis was used to prepare sample size nor replicate number. Rather for the biophysical measurements presented in this manuscript for which this explicit power analysis is not applicable, we focused on biological and technical reproducibility.

**Replicates**

- You should report how often each experiment was performed
- You should include a definition of biological versus technical replication
- The data obtained should be provided and sufficient information should be provided to indicate the number of independent biological and/or technical replicates
- If you encountered any outliers, you should describe how these were handled
- Criteria for exclusion/inclusion of data should be clearly stated
- High-throughput sequence data should be uploaded before submission, with a private link for reviewers provided (these are available from both GEO and ArrayExpress)

A definition for biological and technical replicates is provided in Materials and Methods, at the end of section “Single particle tracking and data analysis”. When applicable, the total number of trajectories in a condition is printed within the corresponding figure [Fig2E, 3C, 4B, 5C, 6D&F]. The total number of molecules in TIRF experiments is printed in the corresponding figure legend [Fig1, Fig1S1]. For optical tweezers measurements, the total number of biological and technical replicates can be found in Table 3, referenced in the Materials and Methods section. For TIRF data, technical replicate information can be found in the corresponding figure legend [Fig1, Fig1S1].

For exclusion/inclusion criteria of single particle trajectories as well as for how outlier trajectories were handled see Materials and Methods section “Single particle tracking and data analysis” as well as Table 3.

Please outline where this information can be found within the submission (e.g., sections or figure legends), or explain why this information doesn’t apply to your submission:

**Statistical reporting**

- Statistical analysis methods should be described and justified
- Raw data should be presented in figures whenever informative to do so (typically when N per group is less than 10)
- For each experiment, you should identify the statistical tests used, exact values of N, definitions of center, methods of multiple test correction, and dispersion and precision measures (e.g., mean, median, SD, SEM, confidence intervals; and, for the major substantive results, a measure of effect size (e.g., Pearson's r, Cohen's d)
- Report exact p-values wherever possible alongside the summary statistics and 95% confidence intervals. These should be reported for all key questions and not only when the p-value is less than 0.05.

Please outline where this information can be found within the submission (e.g., sections or figure legends), or explain why this information doesn’t apply to your submission:

All statistical tests are described in figure captions when they are not described in the results text.

Fig1F,G (caption), Fig2D (Multiple linear fits shown, p-value and Pearson's r criteria for all fits used in diffusion coefficient calculations is summarized in Table 3 and discussed in Materials and Methods section “Single particle tracking and data analysis”), Fig3A (caption), Fig3C (printed in the figure), Fig3D (Results section text, error is propagated using a method printed in Table 3, n-values printed in Fig3C), Fig3E (caption), Fig4A,B (printed in the figure, discussed in caption), Fig4C (N-values have already been presented either in other panels of this figure or in the previous figure), Fig5C (printed in the figure), Fig6D, E, F (printed in the figure).

Summary provided in Table 3

(For large datasets, or papers with a very large number of statistical tests, you may upload a single table file with tests, Ns, etc., with reference to sections in the manuscript.)

**Group allocation**

- Indicate how samples were allocated into experimental groups (in the case of clinical studies, please specify allocation to treatment method); if randomization was used, please also state if restricted randomization was applied
- Indicate if masking was used during group allocation, data collection and/or data analysis

Please outline where this information can be found within the submission (e.g., sections or figure legends), or explain why this information doesn’t apply to your submission:

Does not apply to this study because experiments were all *in-vitro*.

**Additional data files (“source data”)**

- We encourage you to upload relevant additional data files, such as numerical data that are represented as a graph in a figure, or as a summary table
- Where provided, these should be in the most useful format, and they can be uploaded as “Source data” files linked to a main figure or table
- Include model definition files including the full list of parameters used
- Include code used for data analysis (e.g., R, MatLab)
- Avoid stating that data files are “available upon request”

Please indicate the figures or tables for which source data files have been provided:

**Source data:**

Figure 1 – Source data 1

Numerical data and statistics underlying panel F and G

Figure 1 – Source data 2

Gel images (Coomassie and Cy3 scans) shown in panel B

Figure 1–figure supplement 1– source data 1

Gel images (Coomassie and Cy3 scans) shown in panels A and B

Figure 1–figure supplement 1– source data 2

Excel file corresponding to panels C, F, and H

Figure 2 – Source data 1

Data underlying panel D and E

Figure 2 – Source data 2

Uncropped kymograph Tiff image from panel C

Figure 2–figure supplement 1– source data 1

Raw scans and kymograph tiff files

Figure 3 – Source data 1

Data underlying panels A, C, D, and E

Figure 4 – Source data 1

Data underlying panels A, B, and C

Figure 4–figure supplement 1– source data 1

Gel images (Coomassie and Cy3 scans)

Figure 5 – Source data 1

All colocalization events with classifications indicated

Figure 6 – Source data 1

Data underlying panels B, C, and E

Figure 6–figure supplement 1– source data 1

Gel images (Coomassie and Cy3 scans)

Figure 6–figure supplement 1– source data 2

Data underlying panel B and D

For optical tweezers data, **raw data** has been uploaded to Dryad in the form of a Matlab structured array. Optical tweezers data is originally in the form of .h5 files, however due to the large size of these files, a more accessible form of the raw data has been provided. <https://datadryad.org/stash/share/9y48eaKB3mrVSSRAzs_ehAq9nudbKkxizDgPbKdZ7c4>

Select **Matlab codes and Jupyter Notebook files** used to analyse raw optical tweezers data have been provided. All Matlab codes used to generate the main text figures related to optical tweezers data have been provided. Please visit: <https://github.com/ccarcam1/SWR1_1D_Diffusion_Publication>
